# In vitro and in silico studies of terpenes, terpenoids and related compounds with larvicidal and pupaecidal activity against *Culex quinquefasciatus* Say (Diptera: *Culicidae*)

**DOI:** 10.1186/s13065-018-0425-2

**Published:** 2018-05-10

**Authors:** S. Andrade-Ochoa, J. Correa-Basurto, L. M. Rodríguez-Valdez, L. E. Sánchez-Torres, B. Nogueda-Torres, G. V. Nevárez-Moorillón

**Affiliations:** 1 Facultad de Ciencias Químicas, Universidad Autónoma de Chihuahua, Circuito Universitario S/N, Campus Universitario II., Chihuahua, Chihuahua Mexico; 2 Escuela Nacional de Ciencias Biológicas, Instituto Politécnico Nacional, Prolongación de Carpio y Plan de Ayala S/N. Col. Santo Tomas, 11340 México, DF Mexico; 3 Escuela Superior de Medicina, Instituto Politécnico Nacional, Plan de San Luis y Díaz Mirón s/n, Col. Casco de Santo Tomas, Delegación Miguel Hidalgo, C.P. 11340 México, DF Mexico

**Keywords:** QSAR, Essential oils, Larvicidal activity, Sterol carrier protein-2, Terpenes

## Abstract

**Background:**

In order to develop new larvicidal agents derived from phytochemicals, the larvicidal activity of fifty molecules that are constituent of essential oils was evaluated against *Culex quinquefasciatus* Say. Terpenes, terpenoids and phenylpropanoids molecules were included in the *in vitro* evaluation, and QSAR models using genetic algorithms were built to identify molecular and structural properties of biological interest. Further, to obtain structural details on the possible mechanism of action, selected compounds were submitted to docking studies on sterol carrier protein-2 (SCP-2) as possible target.

**Results:**

Results showed high larvicidal activity of carvacrol and thymol on the third and fourth larval stage with a median lethal concentration (LC_50_) of 5.5 and 11.1 µg/mL respectively. Myrcene and carvacrol were highly toxic for pupae, with LC_50_ values of 31.8 and 53.2 µg/mL. Structure–activity models showed that the structural property π-bonds is the largest contributor of larvicidal activity while ketone groups should be avoided. Similarly, property–activity models attributed to the molecular descriptor LogP the most contribution to larvicidal activity, followed by the absolute total charge (*Qtot*) and molar refractivity (*AMR*). The models were statistically significant; thus the information contributes to the design of new larvicidal agents. Docking studies show that all molecules tested have the ability to interact with the SCP-2 protein, wherein α-humulene and β-caryophyllene were the compounds with higher binding energy.

**Conclusions:**

The description of the molecular properties and the structural characteristics responsible for larvicidal activity of the tested compounds were used for the development of mathematical models of structure–activity relationship. The identification of molecular and structural descriptors, as well as studies of molecular docking on the SCP-2 protein, provide insight on the mechanism of action of the active molecules, and the information can be used for the design of new structures for synthesis as potential new larvicidal agents.

**Electronic supplementary material:**

The online version of this article (10.1186/s13065-018-0425-2) contains supplementary material, which is available to authorized users.

## Introduction

More than half of the global human population is exposed to the risk of infection spread by mosquitoes; including *Culex* spp., *Anopheles* spp. and *Aedes* spp. that are considered a public health problem, sin are vectors of pathogenic parasites. Lymphatic filariasis uses *Culex quinquefasciatus* Say (Diptera: *Culicidae*) as vector; it is one of the leading causes of global morbidity, with close to 150 million infected, especially in tropical climates [1]. *Culex quinquefasciatus* is present in most tropical regions of the world; it is commonly found in many urban areas and has been reported as resistant to registered insecticides [2].

The control of mosquito larvae and pupae currently relies on the use of synthetic chemical insecticides [3]. However, prolonged use of these synthetic pesticides has caused numerous problems, such as the development of resistance [4], undesirable effects on non-target organisms, effects on wildlife, damage to human health and other negative impacts on the environment [5–7]. Several studies have searched for natural products derived from plants as possible mosquito control environmentally-friendly strategy; reports include the larvicidal action of essential oils (EOs) and their constituents [8, 9]. EOs can be alternative pest control agents, because some of their compounds have proven to be highly selective, easily removable, biodegradable, with low or no toxicity against mammals and are effective against a full spectrum of mosquito pests [10, 11]. Also EOs are characterized by reduced effects on non target organisms and minimal environmental persistence [12]. With few exceptions, some of the purified terpenoid constituents of EOs are moderately toxic to mammals, but the oils themselves or their compounds are mostly non toxic to mammals, birds, and fish [12].

EOs are heterogeneous mixtures of organic chemical compounds [13] mainly terpenoids and phenylpropanes, but low molecular weight aliphatic compounds, acyclic esters or lactones may also be present [14]. The EOs chemical composition is affected by diverse factors, including plant species and subspecies, geographical location, harvested time, the part of the plant used and the extraction methods employed to obtain the EO [15]. In spite of several studies on the larvicidal activity of EOs and their constituents, little is known on the mechanism of action exerted by terpenoids and phenylpropanoids on mosquito larvae. This has motivated the study of the molecular properties, reactivity or structural modulation of essential oil chemical components in order to minimize synthetic and biological evaluation effort for the development of new compounds with potential larvicidal activity.

Computer assisted prediction of the biological activity of specific chemical compounds considering their chemical structure is now a common technique used in drug discovery [16, 17]. Quantitative structure–activity relationship (QSAR) and quantitative property–activity relationship (QPAR) studies can provide information to understand the relationship between molecule’s chemical structure and biological activity [18]. Also, molecular docking is an in silico technique used to estimate the strength of the protein–ligand interaction, to determine biding poses and free energy values [19]. Docking describe ligand binding to a receptor through noncovalent interactions which is commonly used to explore the ligand recognition on targets for new drug development [20].

This article describes the larvicidal activity of fifty compounds against larvae and pupae of *Culex quinquefasciatus* (Diptera: *Culicidae*). Terpenes, terpenoids and others related compounds constituents of different EOs were evaluated in this work. Likewise, the present work reports the theoretical characterization of the molecular and electronic properties of experimentally tested molecules. QSAR/QPAR models and docking studies are also included to emphasize the molecular and structural properties that are essential in the larvicidal activity.

## Materials and methods

### Compounds tested

**Fig. 1:**
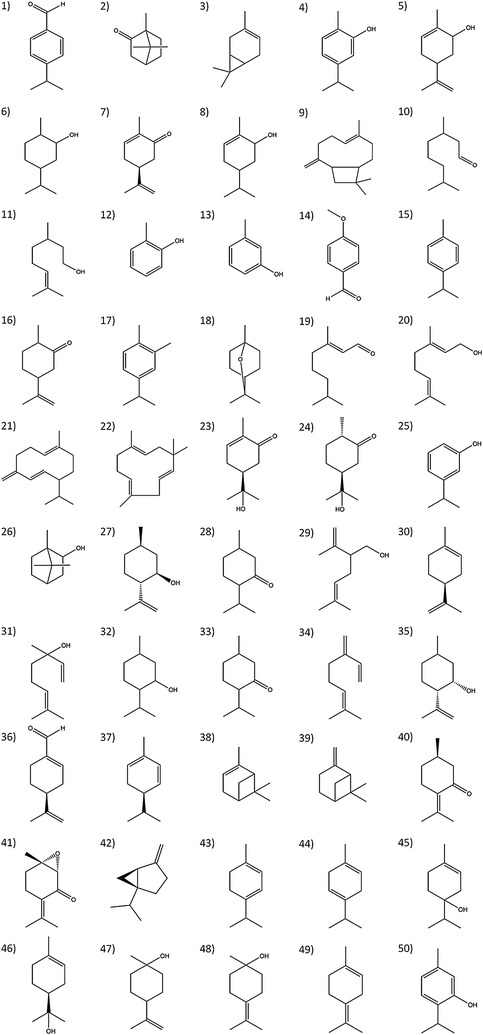
(1) *p*-Anisaldehyde, (2) Canphor, (3) (3) Carene, (4) Carvacrol, (5) Carveol, (6) Carvomenthol, (7) Carvone, (8) Carvotanacetol, (9) β-Caryophyllene, (10) Citronellal, (11) β-Citronellol, (12) m-Cresol, (13) *o*-Cresol, (14) Cuminaldehyde, (15) *p*-Cimene, (16) t-Dihydrocarvone, (17) 3,4-Dimethylcumene, (18) Eucalyptol, (19) Geranial, (20) Geraniol, (21) Germacrene-D, (22) α-Humulene, (23) Hydrocarvone, (24) Hydrodihydrocarvone, (25) 3-Isopropylphenol, (26) Isoborneol, (27) Isopulegol, (28) t-Isopulegone, (29) Lavandullol, (30) Limonene, (31) Linalool, (32) Menthol, (33) Menthone, (34) Myrcene, (35) Neoisopulegol, (36) Perillaldehyde, (37) β-Phellandrene, (38) α-Pinene, (39) β-Pinene, (40) Pulegone, (41) Rotundifolone, (42) Sabinene (43) α-Terpinene, (44) γ-Terpinene, (45) 4-Terpineol, (46) α-Terpineol, 47) β-Terpineol, 48) γ-Terpineol, (49) Terpinolene, (50) Thymol

Fifty compounds were evaluated to determine their larvicidal activity against larvae (stair III and IV) and pupae of *Culex quinquefasciatus* Say (Diptera: *Culicidae*). Compounds were purchased from a Sigma-Aldrich (St. Louis, MI, USA) distributor, and its chemical structure is shown in Fig. 1.

### Insect cultures and rearing conditions

Larvae of *Cx. quinquefasciatus* were collected from water tanks in the Sanctorum Cemetery in Mexico City, Mexico (19°27′17″N, 99°12′47″W) and identified using Harwood and James descriptions [21]. Groups of 50 individuals of first and second instar larvae were placed in glass bottles with purified water, maintained at 26 ± 2° C with a natural photoperiod and supplied with 3:1 powdered mixture of dog food and baking powder. The third instar emerging larvae were then separated by groups of 10 individuals in 100 mL tubes with distilled water [22].

### Larvicidal activity bioassays and statistical analysis

Bioassays were done according to the World Health Organization (WHO) protocol with few modifications [23]. Third and fourth instar larvae as well as pupae, were used for testing. Five groups of 20 larvae were isolated in beakers of 250 mL, exposed to different concentrations of the tested compounds and maintained in starvation throughout the experimental period; the surviving larvae were counted in order to record larval mortality. The compounds were diluted in dimethyl sulfoxide (DMSO) (Sigma, 472301) before being added to the aqueous medium which contained the larvae. Temephos H at 0.1 ppm (commercial concentration) was used as a standard for comparison. Larvae were considered dead if they were immobile and unable to reach the water surface [24]. Lethal concentrations (LC_50_) was calculated using Probit analysis. Data were processed using MS Excel 2010 and SAS v. 9 (Proc Probit) computer programs.

### DFT study and descriptors calculations

Computational studies were carried out using the Spartan 03 [25] and Gaussian 09 quantum chemistry computer programs [26]. The molecular structures were analyzed by a conformational analysis of each molecule in gas phase using the mechanics force field SYBYL [27]. The minimum energy conformation was selected in order to obtain the geometry optimization using the density functional theory (DFT). The equilibrium geometries of the molecules in the electronic ground state were determined with the Becke three-parameter hybrid functional combined with Lee–Yang–Parr correlation functional (B3LYP) [28, 29]. The basis set 6-311G(d,p) was used for the geometry optimization and vibrational frequency calculations and the 6-311+G(d,p) was applied for vertical excitation energy calculations [30–32]. Analytical frequency calculations were carried out, where the absence of imaginary frequencies confirmed that the stationary points correspond to the global minima of the potential energy hypersurfaces.

The Koopmans theorem [33] was applied for calculations of the chemical reactivity descriptors such as: the ionization potential (*I*), electron affinity (*A*), electronegativity (*χ*), chemical potential (*μ*), hardness (*ɳ*), softness (*σ*), global electrophilicity (*ω*), as well as the electronic parameters of, E_HOMO_ (energy of highest occupied molecular orbital), E_LUMO_ (energy of the lowest unoccupied molecular orbital) and band gap (GAP_E_) were calculated. All molecules were analyzed in the gas and aqueous phase. The polarizable continuum model (PCM) was used to model the solvent effects [34].

Structure, constitutional, physicochemical and topological descriptors were generated using Dragon 5.0 software [35] using the optimized structure in the aqueous phase.

### Structure–property–larvicidal activity models

QSAR/QPAR studies was carried out using all biological activities obtained in vitro and the calculated theoretical descriptors; the analysis was carried out using genetic algorithms with the Mobydigs Software [36]. The quality of the model was considered statistically satisfactory based on the determination coefficient (R^2^), leave-one-out cross-validated explained variance (Q^2^), standard deviation (s) and the ANOVA (F) of the model.

### Molecular docking studies on protein SCP-2

The sequence of sterol carrier protein (SCP-2) of *Cx. quinquefasciatus* (GenBank: AAO43438.1) was obtained from the database of the National Center for Biotechnology Information (NCBI). The protein was modeled through Swiss-Model server [37, 38], using as template the sterol carrier protein of *Aedes aegypti* (PDB: 1PZ4) [39] reported in the RCSB Protein Data Bank. The final model was subjected to Ramachandran analysis using the Rampage server [40]. Docking analysis was done using the AutoDock4 software [41]. For the docking the active site was defined considering the residues within a grid of 60 A° × 60 A° × 60 A° centered in the active site, with an initial population of 100 randomly placed individuals and a maximum number of 1.0 × 10^7^ energy evaluations. Active site was determined under the description made by Dyer et al. [39]. Compounds for docking were drawn in Gauss view before docking, the compounds were subjected to energy minimization using the hybrid functional B3LYP with a 6, 311G(d,p) basis set. The *Kd* and ΔG (Kcal/mol) values were obtained from the conformation with the lowest minimum free energy of the ligand coupled on the protein targets. The figures were prepared with ChemBioOffice [42] for the structures and Chimera [43] for the proteins and ligands.

## Results and discusion

### Larvicidal activity and quantitative structure–larvicidal activity relationship

Chemical compounds known to be constituents of EOs demonstrated larvicidal activity against III and IV stairs of *Cx. quinquefasciatus*; activity against pupae was moderate, with higher concentrations of the compounds required to reach LC_50_; LC_50_ values as shown in Table 1. In all experiments, 100% of the larvae remained active in the negative control; DMSO larvicidal activity was also determined, and concentration of 1000 µg/mL had no larvicidal effect; therefore, larvicidal activity can be attributed entirely to the compounds, and not the solvent used.

**Table 1:** Larvicidal activity of the terpenes, terpenoids and related compounds against *Cx. quinquefasciatus*

	Assays		Larvicidal activity (µg/mL)		
			III	IV	Pupaes
	Molecules	Classification	LC_50_	LC_50_	LC_50_
1	*p*-Anisaldehyde	Benzaldehyde	18.0 (15.5–20.4)	18.8 (16.9–20.6)	96.4 (92.5–100.2)
2	Canphor	Bicyclic monoterpenoid	22.3 (21.6–23.9)	25.8 (23.6–27.9)	245.1 (234.6–255.5)
3	3-Carene	Bicyclic monoterpene	24.7 (23.7–25.7)	25.5 (24.3–26.7)	105.5 (101.8–109.1)
4	Carvacrol	Cyclic monoterpenoid	5.5 (5.28–5.72)	7.7 (7.3–8.1)	53.2 (51.8–54.5)
5	Carveol	Cyclic monoterpenoid	103.0 (99.4–109.9)	104.6 (102.0–107.2)	249.0 (241.8–256.1)
6	Carvomenthol	Cyclic monoterpenoid	198.2 (183.69–212.71)	219.8 (206.6–232.9)	452.2 (435.2–469.1)
7	(+)-Carvone	Cyclic monoterpenoid	150.2 (149.0–151.4)	150.2 (145.5–154.8)	500.6 (495.0–506.1)
8	Carvotanacetol	Cyclic monoterpenoid	152.3 (148.2–156.8)	198.3 (192.1–204.44)	245.1 (238.1–252.0)
9	*β*-Caryophyllene	Bicyclic sesquiterpene	45.6 (43.8–47.2)	47.7 (42.2–52.9)	222.3 (216.8–27.7)
10	Citronellal	Acyclic monoterpenoid	105.3 (98.3–102.3)	124.9 (123.2–125.6)	549.2 (557.35–565.5)
11	*β*-Citronellol	Acyclic monoterpenoid	90.4 (88.9–91.9)	94.8 (93.4–95.2)	203.1 (198.44–207.76)
12	*m*-Cresol	Phenolic derivative	60.0 (58.8–61.2)	60.6 (59.3–61.9)	107.7 (104.94–110.4)
13	*o*-Cresol	Phenolic derivative	54.8 (53.6–56.0)	54.4 (53.8–54.0)	105.6 (103.4–107.7)
14	Cuminaldehyde	Benzaldehyde	23.0 (22.0–24.0)	23.9 (22.0–25.8)	95.4 (91.1–99.6)
15	*p*-Cimene	Cyclic monoterpene	23.1 (22.3–24.9)	24.0 (23.8–26.2)	306.3 (298.4–314.1)
16	trans-Dihydrocarvone	Cyclic monoterpene	345.0 (340.8–350.1)	361.3 (346.2–366.4)	708.6 (698.1–719.1)
17	3,4-Dimethylcumene	Phenolic derivative	35.6 (33.5–37.7)	47.7 (46.2–49.2)	105.5 (101.9–109.1)
18	Eucalyptol	Bicyclic monoterpenoid	48.0 (47.9–49.1)	44.4 (43.3–45.5)	92.9 (86.2–99.6)
19	Geranial	Acyclic monoterpenoid	52.2 (51.1–53.3)	53.4 (49.9–56.8)	193.9 (186.8–200.9)
20	Geraniol	Acyclic monoterpenoid	20.4 (19.78–21.02)	20.4 (19.4–21.3)	104.6 (101.9–107.2)
21	Germacrene-D	Sesquiterpene	45.4 (44.3–46.6)	45.6 (46.71–47.49)	229.0 (222.7–235.2)
22	α-Humulene	Bicyclic sesquiterpene	100.5 (98.2–102.7)	101.8 (100.0–103.5)	508.3 (497.17–519.43)
23	Hydrocarvone	Cyclic monoterpene	1351.6 (1228.68–1474.5)	1470.9 (1347.9–1592.9)	> 2000
24	Hydrodihydrocarvone	Cyclic monoterpenemonoterpene	1416.5 (1152.4–1680.1)	1628.2 (1364.6–1889.3)	> 2000
25	3-Isopropylphenol	Cyclic monoterpene	21.3 (20.9–21.6)	23.1 (21.2–24.9)	100.2 (96.4–104.4)
26	Isoborneol	Bicyclic monoterpenoid	91.9 (89.7–94.0)	97.1 (94.1–100.1)	206.1 (199.7–213.5)
27	Isopulegol	Cyclic monoterpene	247.4 (234.4–250.9)	297.3 (290.2–304.3)	610.8 (604.6–616.9)
28	trans-Isopulegone	Cyclic monoterpene	529.1 (510.1–537.1)	538.8 (530.7–546.8)	908.6 (896.2–920.9)
29	Lavandullol	Acyclic monoterpenoid	52.2 (51.0–53.3)	56.5 (53.3–59.9)	238.7 (224.6–252.7)
30	Limonene	Cyclic monoterpene	24.2 (23.4–24.9)	27.3 (23.3–28.2)	98.4 (95.4–101.4)
31	Linalool	Acyclic monoterpenoid	26.8 (26.0–27.5)	30.7 (29.7–31.6)	249.0 (241.8–256.1)
32	Menthol	Cyclic monoterpenoid	443.6 (432.3–443.2)	404.1 (381.1–427.0)	529.1 (521.0–537.1)
33	Menthone	Cyclic monoterpenoid	500.6 (495.0–506.1)	508.9 (500.8–516.9)	878.5 (867.4–889.5)
34	Myrcene	Acyclic monoterpene	19.5 (18.5–20.4)	19.1 (18.0–20.2)	31.8 (30.2–33.2)
35	Neoisopulegol	Cyclic monoterpenoid	458.4 (450.2–466.6)	554.2 (545.6–562.7)	908.6 (896.2–920.9)
36	(−)-Perillaldehyde	Cyclic monoterpenoid	95.9 (94.8–97.0)	115.8 (113.0–118.6)	429.1 (422.9–435.22)
37	Phellandrene	Cyclic monoterpene	490.7 (483.1–498.2)	554.3 (545.8–563.0)	908.6 (896.3–920.9)
38	*α*-Pinene	Bicyclic monoterpene	24.4 (23.2–25.5)	25.5 (22.0–28.97)	98.4 (95.4–101.4)
39	*β*-Pinene	Bicyclic monoterpene	19.6 (18.82–20.38)	24.3 (22.8–25.7)	96.9 (89.9–103.9)
40	(+)–Pulegone	Cyclic monoterpenoid	168.7 (665.8–171.59)	188.1 (185.29–190.91)	496.2 (490.4–501.9)
41	Rotundifolone	Cyclic monoterpenoid	58.9 (57.8–59.9)	62.5 (61.5–63.5)	287.4 (279.4–295.3)
42	Sabinene	Bicyclic monoterpene	53.7 (51.9–55.4)	59.0 (58.3–60.7)	268.0 (262.5–273.0)
43	α-Terpinene	Cyclic monoterpene	13.8 (12.9–14.7)	13.6 (12.8–14.3)	209.5 (204.0–214.9)
44	γ-Terpinene	Cyclic monoterpenemonoterpene	45.4 (44.3–46.5)	56.8 (55.7–57.9)	287.4 (280.2–294.6)
45	*4*-Terpineol	Cyclic monoterpenoid	94.2 (91.1–97.3)	97.7(90.6–104.8)	201.8 (195.6–208.0)
46	*α*-Terpineol	Cyclic monoterpenoid	95.9 (93.8–98.0)	98.4 (95.3–101.4)	206.1 (198.4–213.7)
47	*β*-Terpineol	Cyclic monoterpenoid	101.3 (99.5–103.0)	107.4 (103.9–110.8)	508.3 (497.1–519.43)
48	*γ*-Terpineol	Cyclic monoterpenoid	100.5 (98.3–102.7)	103.6 (100.0–109.9)	4965.5 (4949.1–4981.9)
49	Terpinolene	Cyclic monoterpene	20.4 (19.6–21.2)	18.6 (16.9–20.2)	107.4 (103.9–110.8)
50	Thymol	Cyclic monoterpenoid	11.1 (10.28–11.9)	12.2 (11.7–12.7)	111.4 (108.5–114.2)
Tx	Temephos H	Organophosphorus	2.1 (1.8–2.5)	5.6 (4.1–6.7)	34.0 (29.1–39.0)

In parenthesis, 95% confidence intervals, compounds activity is considered significantly different when the 95% CI fail to overlap

EOs are aromatic extracts obtained from plant material that are complex mixtures of volatile secondary metabolites [44]. Some of the compounds present in EOs are terpenes (molecules formed of isoprene units) [45], terpenoids (terpenes with oxygen on its structure) [45] and phenylpropanoids [47]. In the present report, carvacrol and thymol (terpenoids found mainly in the EO of oregano) were the most active molecules with a LC_50_ of 7.7 and 8.4 μg/mL respectively, against larvae at fourth stage. Myrcene presented a relevant activity against pupae with a LC_50_ of 31.8 μg/mL. Cheng et al. reported the results of screening EOs and suggested that oils with LC_50_ values >  100 ppm should not be considered active, whereas those with LC_50_ values < 50 ppm could be regarded as highly active [48]. Our results agree with reports of the larvicidal activity of constituents of oregano EO; the reports demonstrate that these compounds have fumigant and repellent activity [49–53].

In relation to chemical structure and larvicidal activity, results have been grouped considering the main chemical moiety of the tested compounds in monocyclic-terpenes, monocyclic-terpenoids, bicyclic-terpenes and bicyclic-terpenoids, and phenylpropanes. *β*-Caryophyllene, a bicyclic sesquiterpene, showed the lower larvicidal activity with a LC_50_ of 57.7 μg/mL against fourth instar and 222.3 μg/mL against pupae, Doria et al. also report low larvicidal activity of *β*-caryophyllene against *Aedes aegypti* [54]. Sabineno, a bicyclic monoterpene, also had a low activity, with LC_50_ values of 59.0 μg/mL for fourth instar and 258 μg/mL against pupae. *β*-Pinene and 3-carene presented a LC_50_ of 19.6 and 24.7 μg/mL respectively against the fourth stair being the most active of the bicyclic terpenes. Eucalyptol was the bicyclic terpenoid most active against pupae, the only activity lower than 100 μg/mL of all bicyclic compounds evaluated.

Table 2 include the QSAR models of larvicidal activity against the fourth instar with greater statistical significance. The models were built based on structural descriptors; models 1 and 2 describe the biological activity of the fifty molecules evaluated, and includes the number of total tertiary carbons (sp^3^) (*nCt*) and the number of non-aromatic conjugated carbons (sp^2^) (*nCconj*) as the structural descriptors that contribute the most to the biological activity, whereas the number of ketones (*nRCO*) and number of ethers (*nROR*) showed an inverse relationship with larvicidal activity. The structural descriptors that were less significant, including molecules without benzene ring (models 1 and 2, Table 2) were present in the tested molecules with the lowest biological activity. Sabinene and β-caryophyllene are examples of molecules with no benzene rings and presence of ketone groups. In fact the keto group reduces the activity of carvone more than a half as compared to limonene, which does not have keto groups in its structure.

**Table 2:** Summary of the statistics quantitative structure–larvicidal activity relationship models for activity against fourth instar of *Cx. quinquefasciatus*

Statistical parameter	IV instar					
	Model 1	Model 2	Model 3	Model 4	Model 5	Model 6
n	50	50	47	47	39	39
Q^2^	0.793	0.75.34	0.781	0.759	0.851	0.832
R^2^	0.828	0.78.73	0.881	0.858	0.965	0.957
F	14.5	11.1	21.8	21.3	49.2	39.4
s	0.291	0.301	0.231	0.234	0.137	0.152

Descriptors	Contributions					
	Model 1	Model 2	Model 3	Model 4	Model 5	Model 6
*nCt*	0.0679	*WC*	*WC*	*WC*	*WC*	*WC*
*nCconj*	*0.0631*	0.04241	0.052	0.0444	0.3304	0.3606
*nR* = *Cp*	*WC*	0.0803	*WC*	*WC*	*WC*	*WC*
*nRCO*	− *0.5006*	− 0.5641	− 0.491	− 0.48	− 0.7285	− 0.6265
*nROR*	− *0.34331*	*WC*	− 0.2618	− 0.2579	− 0.503	− 0.5205
*nArOH*	*WC*	0.1229	0.1518	*WC*	0.6552	0.6582
*nOH*	*WC*	*WC*	*WC*	0.0187	*WC*	*WC*
Intercept	− 1.5373	− 1.644	− 1.6531	− 1.6723	− 2.80322	− 2.8386

n, number of systems evaluated; Q^2^, the square of the coefficient of cross-validation; R^2^, the square of the correlation coefficient; s, standard deviation; F, Fisher statistic; *WC*, without contribution; *nCt*, number of total tertiary C (sp^3^); *nCconj*, number of non-aromatic conjugated C (sp^2^); *nR* = *Cp*, number of terminal primary C (sp^2^); *nRCO*, number of ketones (aliphatic); nROR, number of ethers; *nArOH*, number of phenolic groups; *nOH*, number of a hydroxyls

Models 3 and 4 (Table 2) were constructed based on the larvicidal activity of 47 evaluated molecules, excluding the sesquiterpenes *β*-caryophyllene (**9**), germacrene (**21**) and *α*-humulene (**22**) from the analysis. The models showed the same relationship with the *nCconj*, *nRCO*, *nROR* descriptors and the number of phenolic groups (*nArOH*) and the number of hydroxyl groups (*nOH*) as descriptors directly related to the biological activity. This is consistent with the most biologically-active molecules: carvacrol and thymol. In monocyclic terpenoids and monocyclic terpenes, increasing the number of double bonds also increased the larvicidal activity. Menthol has a LC_50_ of 38.1 μg/mL against fourth instar larvae, while thymol had an activity of 12.2 μg/mL. The structural difference between these two compounds is the phenolic group in thymol as compared to menthol that only has the hydroxyl group; *p*-Cymene has the benzene group without hydroxyl group with an activity of 24.0 μg/mL; this demonstrate the importance of the phenolic group in the larvicidal activity. Carvacrol, an isomer of thymol, has a LC_50_ of 7.7 μg/mL; therefore, the position of the hydroxyl group plays an important role in the larvicidal activity.

For acyclic terpenes and terpenoids, higher larvicidal activity was observed in compounds with a higher number of double bonds and increased lipophilicity. Ketone acyclic terpenes were the compounds with lowest larvicidal activity; substitution of the ketone group by the hydroxyl group increased the biological activity considerably. Citronellol was the alcohol terpene with lower activity against the fourth instar and pupae. Geraniol has one double bond more than citronellol, and this structural difference increase the larvicidal activity. Linalool also presents one double bond more than citronellol, and this differential structure is reflected in an increase in larvicidal activity, however the position of the hydroxyl group changes from a primary to a secondary alcohol; this difference could be responsible for the lower biological activity shown. On the other hand, myrcene, an acyclic molecule with no oxygen in its structure, has the highest larvicidal activity and is the only compund with significant activity against pupae, with a LD_50_ of 19.1 μg/mL against the fourth instar larvae and 31.8 μg/mL against pupae. Myrcene has three double bonds in its structure, and since the lipophilicity is increased in the absence of oxygen, these is an important trait for their potential activity. Accordingly, Lucia et al. consider that the octanol water partition coefficient (*LogP*) is an important molecular property in the larvicidal activity of monoterpenes [55].

**Fig. 2:**
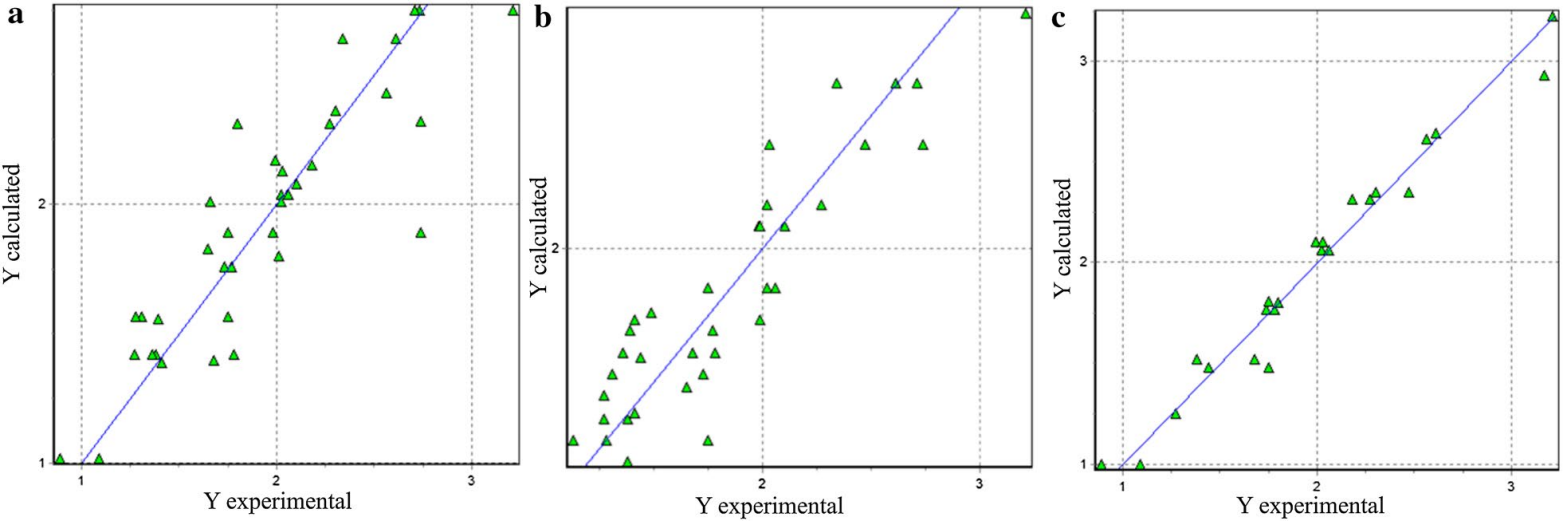
Predicted versus experimental larvicidal activity from structural–activity relationship models. **a** Model 1, **b** model 3, **c** model 5

In models 5 and 6, the sesquiterpenes and all acyclic monotepenes were excluded. The relations of the descriptors are maintained although their values increase considerably, demonstrating that *nRCO* and *nROR* obstruct the activity of monoterpenes, so that in order to potentiate the activity of the compounds as larvicides agents, these functional groups must be avoided. On the other hand, the *nArOH* excels on the *nCconj* as the descriptor of greatest contribution in larvicidal activity, an issue discussed previously. The values of structural descriptors for each target system are confined in Table 3. A plot of the predicted activity versus experimental activity for molecules using a training set for structure–activity relationship models is shown in Fig. 2. Experimental and predicted LogLC_50_ values are shown in Additional file 1: Table S1, while the constitutional descriptors can be observed in Additional file 1: Table S2.

**Table 3:** Structural descriptors calculated

Mol.	*nCs*	*nCt*	*nCconj*	*nR* = *Cp*	*nR* = *Cs*	*nR* = *Ct*	*nRCO*	*nArOH*	*nOH*	*nHDon*	*nHAcc*
1	0	0	1	0	0	0	0	0	0	0	2
2	3	1	0	0	0	0	1	0	0	0	1
3	2	2	0	0	1	1	0	0	0	0	0
4	0	1	0	0	0	0	0	1	0	1	1
5	3	1	0	1	1	2	0	0	1	1	1
6	4	3	0	0	0	0	0	0	1	1	1
7	2	1	3	1	1	2	1	0	0	0	1
8	3	2	0	0	1	1	0	0	1	1	1
9	4	2	0	1	1	2	0	0	0	0	0
10	3	1	0	0	1	1	0	0	0	0	1
11	3	1	0	0	1	1	0	0	1	1	1
12	0	0	0	0	0	0	0	1	0	1	1
13	0	0	0	0	0	0	0	1	0	1	1
14	0	1	1	0	0	0	0	0	0	0	1
15	0	1	0	0	0	0	0	0	0	0	0
16	3	2	0	1	0	1	1	0	0	0	1
17	0	1	0	0	0	0	0	0	0	0	0
18	4	1	0	0	0	0	0	0	0	0	0
19	2	0	3	0	2	2	0	0	0	0	1
20	2	0	0	0	2	2	0	0	1	1	1
21	4	2	4	1	3	2	0	0	0	0	0
22	4	0	0	0	4	2	0	0	0	0	0
23	2	2	3	0	1	1	1	0	1	1	2
24	3	3	0	0	0	0	1	0	1	1	2
25	0	1	0	0	0	0	0	1	0	1	1
26	4	1	0	0	0	0	0	0	1	1	1
27	4	2	0	1	0	1	0	0	1	1	1
28	3	3	0	0	0	0	1	0	0	0	1
29	2	1	0	1	1	2	0	0	1	1	1
30	3	1	0	1	1	2	0	0	0	0	0
31	2	1	0	1	2	1	0	0	1	1	1
32	4	3	0	0	0	0	0	0	0	1	1
33	3	3	0	0	0	0	1	0	0	0	1
34	2	0	4	2	2	2	0	0	0	0	0
35	4	2	0	1	0	1	0	0	1	1	1
36	3	1	3	1	1	2	0	0	0	0	1
37	2	1	4	0	2	2	0	0	0	0	0
38	2	2	0	0	1	1	0	0	0	0	0
39	3	2	0	1	0	1	0	0	0	0	0
40	3	1	3	0	0	2	1	0	0	0	1
41	3	1	3	0	0	2	1	0	0	0	2
42	3	2	0	1	0	1	0	0	0	0	0
43	2	1	4	0	2	2	0	0	0	0	0
44	2	1	0	0	2	2	0	0	0	0	0
45	3	2	0	0	1	1	0	0	1	1	1
46	3	2	0	0	1	1	0	0	1	1	1
47	4	2	0	1	0	1	0	0	1	1	1
48	4	1	0	0	0	2	0	0	1	1	1
49	3	0	0	0	1	3	0	0	0	0	0
50	0	1	0	0	0	0	0	1	0	1	1

*nCs*, Number of total secondary C (sp^3^); *nCt*, number of total tertiary C (sp^3^); *nCconj*, Number of non-aromatic conjugated C (sp^2^); *nR* = *Cp*, number of terminal primary C (sp^2^); *nR* = *Cs*, number of aliphatic secondary C(sp^2^); *nR* = *Ct*, number of aliphatic tertiary C(sp^2^); *nRCO*, number of ketones (aliphatic); nArOH, number of aromatic hydroxyls; *nOH*, number of a hydroxyls; *nHDon*, number of donor atoms for H-bonds; *nHAcc*, number of acceptor atoms for H-bonds

### Quantitative property–larvicidal activity relationship and DFT study

The models that describe the relationship between the molecular properties and biological activity demonstrate that the octanol–water partition coefficient (*MlogP*) descriptor is the largest contributor to the larvicidal activity. Lucia et al. developed a QSAR model based on six monoterpenes and they found that when vapor pressure and lipophilicity values decreased, the larvicidal activity against *A. aegypti* also diminished. The strong effect of the octanol–water partition coefficient can be explained considering that the main conduit for component entrance to the organism is tactile (external cuticle) [55]. Therefore, the partition occurs between the hydrophilic environment (water) and the lipophilic environment (larval epicuticle); therefore, molecule hydrophobicity plays an important role in the intoxication of the larva [56].

**Fig. 3:**
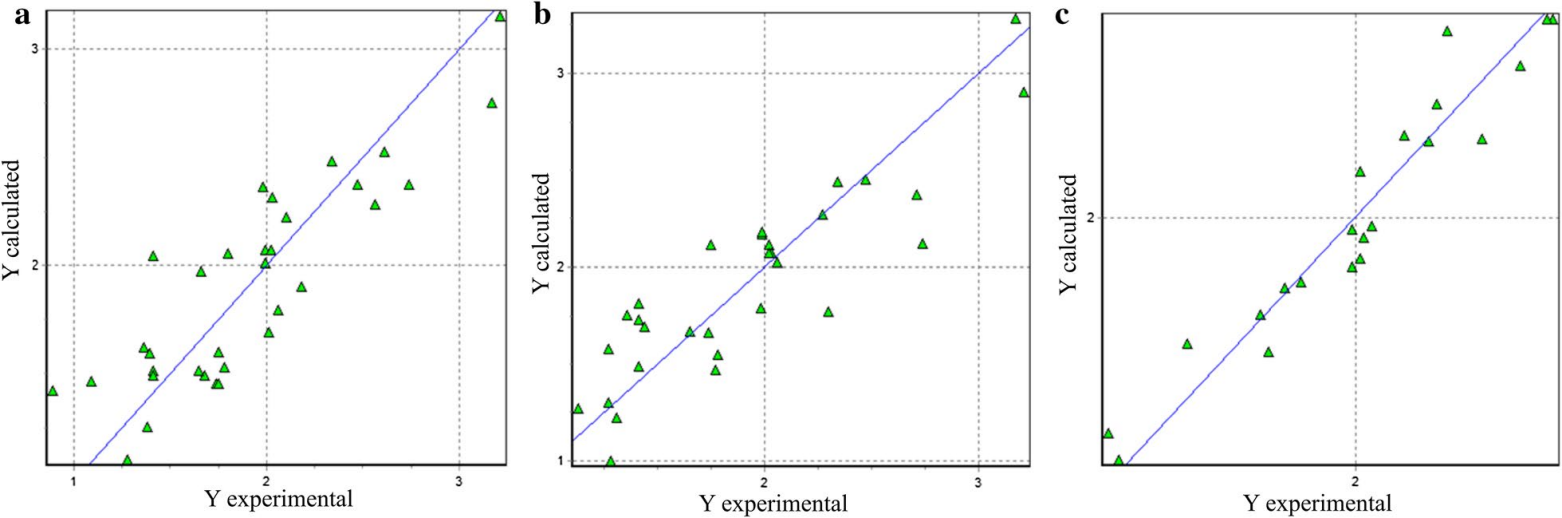
Predicted versus experimental larvicidal activity from property–activity relationship models. **a** Model 1, **b** model 3, **c** model 5

Table 4 includes the QPAR models of larvicidal activity against the fourth instar with greater statistical significance. Like QSAR models, QPAR models 1 and 2 were constricted based on all the evaluated compounds, in the models 3 and 4 the sesquiterpenos were excluded and the models 5 and 6 were constructed excluding sesquiterpenes and acyclic monoterpenes. The predicted activity versus experimental activity for molecules using a training set for structure–activity relationship models is shown in Fig. 3. Experimental and predicted LogLC_50_ values of QPAR models are shown in Additional file 1: Table S3.

**Table 4:** Summary of the statistics quantitative property–larvicidal activity relationship models for activity against fourth instar of *Cx. quinquefasciatus*

Statistical parameter	IV instar					
	Model 1	Model 2	Model 3	Model 4	Model 5	Model 6
n	50	50	47	47	39	39
Q^2^	0.759	0.630	0.761	0.751	0.840	0.818
R^2^	0.829	0.812	0.880	0.880	0.929	0.917
F	20.9	20.2	24.1	23.8	34.3	29.6
s	0.293	0.297	0.022	0.021	0.151	0.162

Descriptors	Contributions					
	Model 1	Model 2	Model 3	Model 4	Model 5	Model 6
*J*	− 2.3271	− 1.6812	*WC*	*WC*	− 0.0638	*WC*
*MlogP*	0.3632	0.3222	*WC*	*WC*	1.5415	1.1347
*TIE*	0.0843	0.0929	0.16824	0.1684	0.2377	0.0467
*AMR*	0.0441	*WC*	*WC*	*WC*	*WC*	*WC*
*Qtot*	*WC*	*WC*	0.4735	0.5324	*WC*	*WC*
*BAC*	*WC*	− 0.0535	*WC*	*WC*	*WC*	*WC*
*Hy*	*WC*	*WC*	− 0.7359	− 0.4735	*WC*	*WC*
*ƞ*	*WC*	*WC*	0.3068	WC	*WC*	*WC*
*m*	*WC*	*WC*	*WC*	0.0927	0.5698	0.6654
*E*_*HOMO*_	*WC*	*WC*	*WC*	WC	0.2377	0.2486
Intercept	− 0.3266	− 0.3421	− 2.3992	− 2.3891	7.8613	4.817

n, number of systems evaluated; Q^2^, the square of the coefficient of cross-validation; R^2^, the square of the correlation coefficient; s, standard deviation; F, Fisher statistic; *WC*, without contribution; *J*, Balaban-like index; *MlogP*, Moriguchi octanol–water partition coeff. (logP); *TIE*, E-state topological parameter; *AMR*, Ghose–Crippen molar refractivity; *Qtot*, total absolute charge; *BAC*, Balaban centric index; *Hy*, hydrophilic factor; *η*, chemical hardness; *E*_*HOMO*_, energy of the HOMO orbital; *m*, dipole moment

The lipophilic character of terpenes and their derivatives have been widely discussed as a key factor in the antimicrobial and larvicidal properties of these compounds [14–16, 44, 45]; however, it does not finish describing their larvicidal behavior. Sesquiterpenes, for example, have high *MlogP* values and are not the most active compounds.

Some QPAR models consider molar refractivity (*AMR*) and absolute total charge (*Qtot*) as descriptors that contribute to larvicidal activity. *Qtot* is a measure of the weak intermolecular interactions which provides information on the electrical charges of the molecules and is considered as the driving force of electrostatic interactions, important for the interaction of the component with its biological target [57]. Myrcene, the most active acyclic terpene, is the terpene with largest number of double bonds, more *MlogP* and lowest *Qtot*, also it had the lowest *AMR*. Molar refractivity (*AMR*) descriptor is related to specific interactions with a target molecule and the electronic effects in the biological–chemical interaction, mainly for allosteric effects of interactions between the ligand-receptor [58]: therefore, it demonstrates the importance of interaction with a specific enzyme, pools of metabolites, or signaling pathways [59]. Hanch and Verma proposed a QSAR model for complex triorganotin with larvicidal activity reported by Eng et al., its models included hydrophobicity (*Hy*) and molar refractivity (*AMR*) as the most important parameters for the description of larvicidal activity [60, 61]. In these results, when *MlogP* was not included in the models the *Hy* presented in inverse relation to the larvicidal activity. The values of molecular and physicochemical descriptors for each compound are included in Table 5.

**Table 5:** Molecular and physicochemical descriptors calculated

Mol.	*Qpos*	*Qneg*	*Qtot*	*Ui*	*Hy*	*AMR*	*TPSA (tot)*	*MlogP*	*AlogP*
1	1.28	− 1.28	2.56	3	− 0.768	39.112	26.3	1.49	1.573
2	1.659	− 1.659	3.318	1	− 0.877	44.492	17.07	2.357	1.936
3	1.413	− 1.413	2.825	1	− 0.96	44.722	0	3.374	2.873
4	1.5	− 1.5	2.999	2.807	− 0.294	46.984	20.23	2.813	3.243
5	1.234	− 1.234	2.468	1.585	− 0.294	47.995	20.23	2.25	2.401
6	1.249	− 1.249	2.497	0	− 0.294	47.445	20.23	2.502	2.779
7	1.665	− 1.665	3.331	2	− 0.877	47.174	17.07	2.153	2.361
8	1.704	− 1.704	3.408	1	− 0.294	48.218	20.23	2.357	2.597
9	1.926	− 1.926	3.853	1.585	− 0.975	62.851	0	4.375	4.297
10	1.288	− 1.288	2.576	1.585	− 0.877	49.297	17.07	2.642	3.019
11	1.791	− 1.791	3.582	1	− 0.294	50.486	20.23	2.749	3.049
12	0.884	− 0.884	1.768	2.807	− 0.158	32.793	20.23	1.859	2.049
13	0.867	− 0.867	1.734	2.807	− 0.158	32.793	20.23	1.859	2.049
14	1.444	− 1.444	2.887	3	− 0.877	46.84	17.07	2.723	2.784
15	1.315	− 1.315	2.631	2.807	− 0.96	45.29	0	3.562	3.51
16	1.283	− 1.283	2.566	1.585	− 0.877	46.298	17.07	2.25	2.401
17	0.905	− 0.905	1.81	2.807	− 0.965	50.331	0	3.854	3.997
18	1.422	− 1.422	2.844	0	− 0.96	43.799	0	4.431	3.077
19	1.283	− 1.283	2.566	2	− 0.877	50.199	17.07	2.545	3.19
20	1.684	− 1.684	3.367	1.585	− 0.294	51.182	20.23	2.642	2.934
21	1.478	− 1.478	2.956	2	− 0.977	70.55	0	4.534	5.135
22	1.446	− 1.446	2.892	2	− 0.977	71.549	0	4.534	5.035
23	1.596	− 1.596	3.191	1.585	− 0.244	49.154	37.3	1.369	1.274
24	1.612	− 1.612	3.223	1	− 0.244	48.278	37.3	1.477	1.313
25	1.06	− 1.06	2.12	2.807	− 0.257	41.943	20.23	2.51	2.757
26	1.157	− 1.157	2.313	0	− 0.294	45.314	20.23	2.502	1.975
27	1.232	− 1.232	2.463	1	− 0.294	47.222	20.23	2.357	2.583
28	1.286	− 1.286	2.571	1	− 0.877	46.52	17.07	2.357	2.597
29	1.344	− 1.344	2.688	1.585	− 0.325	54.812	20.23	2.933	3.105
30	1.438	− 1.438	2.877	1.585	− 0.96	46.48	0	3.267	3.503
31	1.882	− 1.882	3.763	1.585	− 0.294	50.206	20.23	2.642	2.735
32	1.772	− 1.772	3.545	0	− 0.294	47.445	20.23	2.502	2.779
33	1.288	− 1.288	2.575	1	− 0.877	46.52	17.07	2.357	2.597
34	1.526	− 1.526	3.053	2	− 0.96	48.379	0	3.562	3.688
35	1.27	− 1.27	2.54	1	− 0.294	47.222	20.23	2.357	2.583
36	1.231	− 1.231	2.461	2	− 0.877	47.272	17.07	2.153	2.668
37	0.89	− 0.89	1.78	1.585	− 0.96	47.553	0	3.267	3.449
38	1.398	− 1.398	2.796	1	− 0.96	44.722	0	3.374	2.873
39	0.933	− 0.933	1.866	1	− 0.96	43.65	0	3.374	2.927
40	1.224	− 1.224	2.448	1.585	− 0.877	47.129	17.07	2.25	2.752
41	1.236	− 1.236	2.472	1.585	− 0.807	46.637	29.6	1.369	1.824
42	1.47	− 1.47	2.941	1	− 0.96	43.65	0	3.374	2.927
43	1.386	− 1.386	2.772	1.585	− 0.96	47.553	0	3.267	3.449
44	0.892	− 0.892	1.784	1.585	− 0.96	47.553	0	3.267	3.449
45	1.257	− 1.257	2.515	1	− 0.294	48.307	20.23	2.357	2.55
46	1.247	− 1.247	2.494	1	− 0.294	48.461	20.23	2.357	2.415
47	1.288	− 1.288	2.577	1	− 0.294	47.388	20.23	2.357	2.469
48	1.195	− 1.195	2.39	1	− 0.294	48.194	20.23	2.357	2.61
49	1.369	− 1.369	2.738	1.585	− 0.96	47.286	0	3.267	3.643
50	1.523	− 1.523	3.045	2.807	− 0.294	46.984	20.23	2.813	3.243

*Qpos*, total positive charge; *Qneg*, total negative charge; *Qtot*, total absolute charge (electronic charge index-ECI); *Ui*, unsaturation index; *Hy*, hydrophilic factor; *AMR*, Ghose–Crippen molar refractivity; *TPSA*, topological polar surface area; *MlogP*, Moriguchi octanol–water partition coeff.; *AlogP*, Ghose–Crippen octanol–water partition coeff

The quantum-chemical parameters, such as: chemical hardness (*η*), dipole moment (*m*) and energy of the HOMO orbital (*E*_*HOMO*_), were considered as descriptors directly related to biological activity by the models. These descriptors, related to chemical reactivity, are derived from the information provided by molecular orbitals. Some authors have suggested that the presence of a free hydroxyl group and a delocalized electron system in terpenes are critical for their antibacterial activity [62]. This proposal is important when the chemical reactivity of carvacrol and thymol with respect to carvomenthol and menthol is compared. Phenolic group reduces the energy values of the frontier orbitals, whereas the hydroxyl groups by itself increase the *η*, making carvomenthol and menthol less reactive and also less active. However, *η* or chemical softness (*S*) cannot be determinants of biological activity, since *p*-cymene presents these values closer to thymol and carvacrol and yet has less activity than menthol and carvomenthol. Thus, the hydroxyl group alone is also important in the larvicidal activity, a factor considered in the QSAR models. The values of the chemical reactivity descriptors are shown in Table 6.

**Table 6:** Chemical reactivity descriptors calculated

Mol.	*E*_*HOMO*_	*E*_*LUMO*_	*GAP*_*E*_	*I*	*A*	*χ*	*µ*	*n*	*σ*	*m*
1	− 8.954	2.096	11.050	8.954	− 2.096	− 3.429	3.429	5.525	0.181	5.429
2	− 10.343	3.969	14.312	10.343	− 3.969	− 3.187	3.187	7.156	0.140	3.927
3	− 8.971	4.13	13.102	8.973	− 4.13	− 2.421	2.421	6.551	0.153	0.178
4	− 8.351	4.112	12.463	8.351	− 4.112	− 2.119	2.119	6.232	0.16	1.672
5	− 9.356	4.94	14.296	9.356	− 4.94	− 2.207	2.207	7.148	0.139	1.96
6	− 10.751	6.545	17.296	10.751	− 6.545	− 2.102	2.102	8.648	0.115	1.751
7	− 9.308	2.797	12.106	9.308	− 2.797	− 3.255	3.255	6.053	0.165	3.989
8	− 8.945	5.270	14.215	8.945	− 5.270	− 1.837	1.837	7.108	0.140	1.864
9	− 8.646	4.517	13.162	8.646	− 4.517	− 2.064	2.064	6.581	0.152	0.711
10	− 9.080	4.409	13.490	9.080	− 4.409	− 2.335	2.335	6.745	0.148	2.873
11	− 8.808	4.832	13.641	8.808	− 4.832	− 1.988	1.988	6.820	0.147	2.137
12	− 8.545	3.951	12.496	8.545	− 3.951	− 2.297	2.297	6.248	0.160	1.368
13	− 8.481	4.075	12.555	8.481	− 4.075	− 2.202	2.202	6.277	0.159	1.921
14	− 9.180	1.997	11.177	9.180	− 1.997	− 3.591	3.591	5.588	0.179	4.237
15	− 8.542	4.17	12.712	8.542	− 4.17	− 2.186	2.186	6.356	0.157	0.054
16	− 9.491	3.82	13.311	9.491	− 3.82	− 2.835	2.835	6.656	0.15	3.599
17	− 8.432	4.303	12.735	8.432	− 4.303	− 2.064	2.064	6.367	0.157	0.328
18	− 10.288	5.493	15.782	10.288	− 5.493	− 2.397	2.397	7.891	0.127	1.727
19	− 9.05	2.340	11.390	9.050	− 2.346	− 3.360	3.360	5.701	0.18	4.732
20	− 8.859	4.724	13.583	8.859	− 4.724	− 2.068	2.068	6.792	0.147	2.409
21	− 8.268	4.476	12.744	8.268	− 4.476	− 1.896	1.896	6.372	0.157	0.39
22	− 8.704	4.909	13.613	8.704	− 4.909	− 1.897	1.897	6.807	0.147	0.206
23	− 7.752	6.518	14.271	7.753	− 6.518	− 0.617	0.617	7.136	0.14	3.777
24	− 10.387	4.417	14.804	10.387	− 4.417	− 2.985	2.985	7.402	0.135	2.525
25	− 8.523	3.985	12.508	8.523	− 3.985	− 2.268	2.268	6.254	0.159	1.397
26	− 10.727	5.844	16.571	10.727	− 5.844	− 2.441	2.441	8.286	0.121	1.855
27	− 9.46	5.094	14.554	9.461	− 5.094	− 2.183	2.183	7.278	0.137	3.754
28	− 10.473	3.961	14.434	10.474	− 3.961	− 3.256	3.256	7.217	0.138	3.506
29	− 8.98	3.88	12.86	8.98	− 3.88	− 2.55	2.55	6.43	0.16	2.32
30	− 8.745	4.901	13.646	8.746	− 4.901	− 1.922	1.922	6.824	0.146	0.586
31	− 9.082	4.442	13.524	9.082	− 4.442	− 2.320	2.320	6.762	0.148	1.361
32	− 10.918	5.517	16.435	10.918	− 5.517	2.7	− 2.7	8.218	0.121	2.047
33	− 10.711	3.419	14.130	10.712	− 3.419	− 3.645	3.645	7.066	0.141	3.632
34	− 8.519	5.024	13.542	8.519	− 5.024	− 1.747	1.747	6.771	0.148	0.751
35	− 9.605	4.832	14.437	9.605	− 4.832	− 2.386	2.386	7.219	0.138	2.324
36	− 9.442	2.596	12.038	9.442	− 2.596	− 3.422	3.422	6.019	0.166	3.631
37	− 7.764	3.901	11.674	7.764	− 3.901	− 1.926	1.926	5.837	0.171	0.516
38	− 8.695	5.170	13.865	8.695	− 5.170	− 1.763	1.763	6.933	0.144	0.176
39	− 8.695	5.170	13.865	8.695	− 5.170	− 1.763	1.763	6.933	0.144	0.164
40	− 9.146	3.51	12.656	9.146	− 3.51	− 2.818	2.818	6.328	0.158	3.559
41	− 9.391	2.798	12.189	9.392	− 2.798	− 3.296	3.296	6.095	0.164	3.748
42	− 8.885	4.062	12.947	8.885	− 4.062	− 2.411	2.411	6.474	0.154	0.841
43	− 9.001	3.052	12.053	9.001	− 3.052	− 2.975	2.975	6.027	0.165	0.802
44	− 7.64	3.378	11.018	7.641	− 3.378	− 2.131	2.131	5.509	0.181	0.648
45	− 9.355	5.102	14.457	9.355	− 5.102	− 2.126	2.126	7.229	0.138	1.841
46	− 9.016	3.878	12.895	9.016	− 3.878	− 2.569	2.569	6.447	0.155	1.901
47	− 9.892	3.097	12.989	9.892	− 3.097	− 3.397	3.397	6.495	0.153	1.691
48	− 9.371	3.852	13.222	9.371	− 3.852	− 2.759	2.759	6.611	0.151	1.772
49	− 8.475	4.996	13.471	8.475	− 4.996	− 1.739	1.739	6.735	0.148	0.198
50	− 8.325	4.145	12.470	8.325	− 4.145	− 2.09	2.09	6.235	0.161	1.765

*E*_*HOMO*_, energy of the HOMO orbital; *E*_*LUMO*_, energy of the LUMO orbital; *GapE*, E_LUMO_–E_HOMO_; *I*, ionization potential; *A*, electron affinity; *μ*, chemical potential; *χ*, electronegativity; ƞ, Chemical hardness *σ*, chemical softness; *m*, dipole moment

**Fig. 4:**
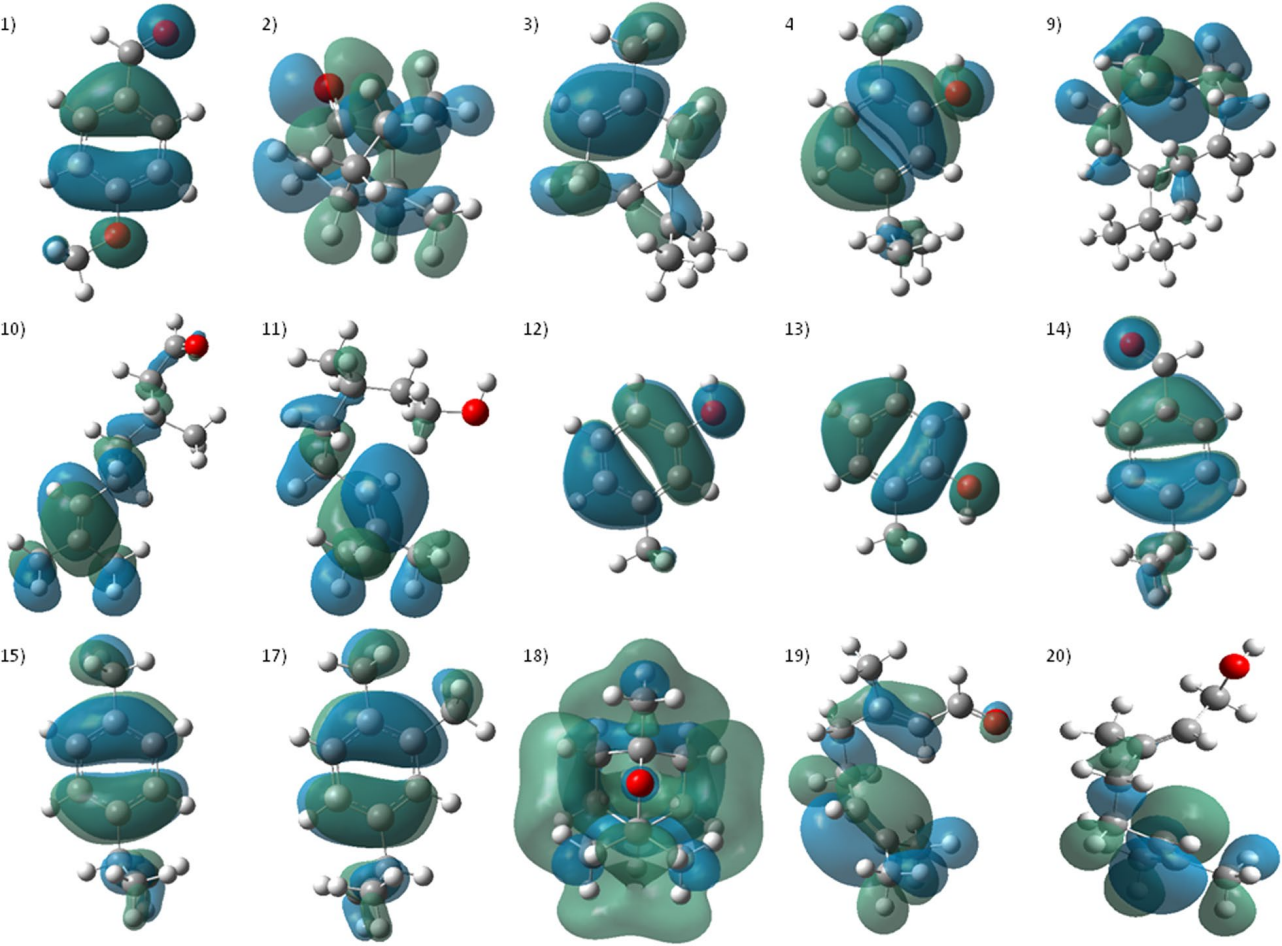
The contour plots of LUMO orbitals of the most active molecules. (1) *p*-Anisaldehyde, (2) Canphor, (3) 3-Carene, (4) Carvacrol, (9) β-Caryophyllene, (10) Citronellal, (11) β-Citronellol, (12) m-Cresol, (13) *o*-Cresol, (14) Cuminaldehyde, (15) *p*-Cimene, (17) 3,4-Dimethylcumene, (18) Eucalyptol, (19) Geranial, (20) Geraniol

**Fig. 5:**
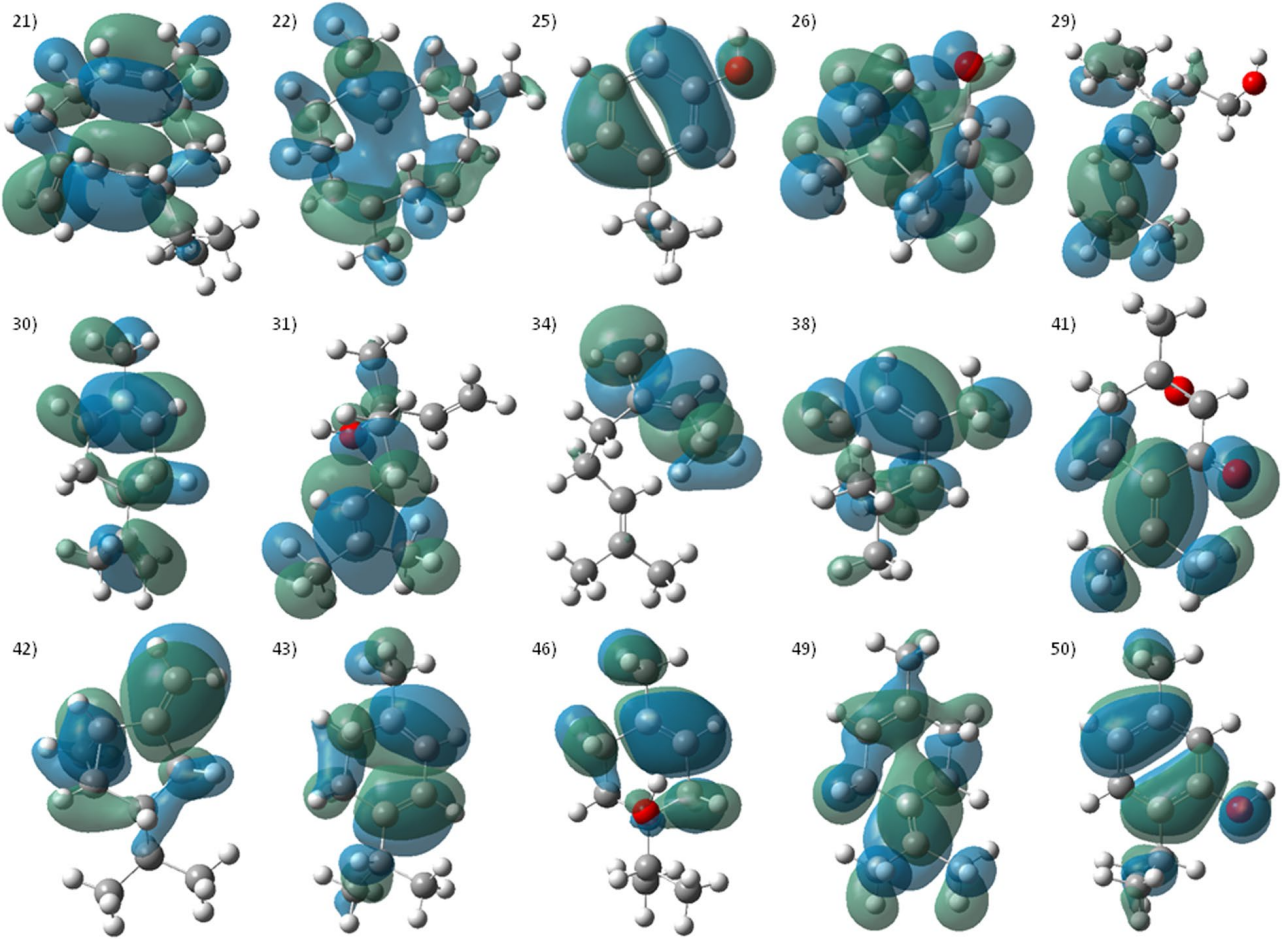
The contour plots of LUMO orbitals of the most active molecules (conti…). (21) Germacrene-D, (22) α-Humulene, (25) 3-Isopropylphenol, 26) Isoborneol, (29) Lavandullol, (30) Limonene, (31) Linalool, (34) Myrcene, (38) α-Pinene, (41) Rotundifolone, (42) Sabinene, (43) α-Terpinene, (46) α-Terpineol, (48) γ-Terpineol, (49) Terpinolene, (50) Thymol

A study conducted with sesquiterpenes found that the repellent activity of these compounds was related primarily to the vapor pressure (*VP*) and electronic properties as LUMO energies [63], so that in their models, repellent activity increased as polarizability decreased, while high LUMO energies maintained a relationship with activity. This relationship is consistent with results applied to monoterpenes and their derivatives. The HOMO orbital is used as an indicator of the highest electron density area, so that these zones exhibit a favorable region to be attacked by electrophiles [64]. Figures 4 and 5 shows the mapping of the HOMO orbitals on the most active molecules, while Additional file 1: Figure S1 shows the mapping of LUMO orbitals.

The models presented demonstrated that the lipophilic character as well as the electronic properties conferred by phenolic groups are important for the larvicidal activity. The models also propose topological descriptors as factors driving the activity, especially when comparing among isomers. The position of the hydroxyl in the thymol molecule favors higher values of the Balaban index (*J*), E-state topological parameter (*TIE*), centralization (*CENT*), variation (*VAR*) and radial centric information index (*ICR*), with respect to carvacrol, as observed in models that incorporate this descriptors. Raising *J* and *TIE* increases the biological activity and explains the difference in activities between carvacrol and thymol. Distance-based index, *J* [65], strongly reflects the molecular branch, based on the sum of the distances from one atom to another in the conformation of the molecule and its value depends on three-dimensional conformation [66], while *TIE* [67, 68] use electronic and topological organization to define the intrinsic atom state and the perturbations of this state induced by other atoms. The values calculated of topological descriptors are listed in Table 7.

**Table 7:** Topological descriptors calculated

Mol.	*J*	*TIE*	*UNIP*	*CENT*	*VAR*	*BAC*	*Lop*	*ICR*	*CSI*	*ECC*	*PHI*
1	2.174	10.042	21	40	14	9	1.261	1.922	100	52	2.185
2	2.396	18.594	16	70	13	17	0.845	1.322	73	36	1.135
3	2.037	10.135	17	46	12	10	0.853	1.571	84	40	1.073
4	2.396	14.877	21	73	14	18	1.16	1.868	99	52	2.3
5	2.396	18.074	21	73	14	18	1.16	1.868	99	52	2.504
6	2.396	19.928	21	73	14	18	1.16	1.868	99	52	2.941
7	2.396	18.471	21	73	14	18	1.16	1.868	99	52	2.281
8	2.396	18.314	21	73	14	18	1.16	1.868	99	52	2.717
9	2.059	19.097	29	144	20	17	0.788	1.531	148	72	2.328
10	3.1	25.28	26	102	22	29	2.187	2.231	122	70	5.82
11	3.1	24.613	26	102	22	29	2.187	2.231	122	70	6.24
12	2.231	6.729	13	18	6	5	0.875	1	54	28	1.31
13	2.279	7.081	12	24	6	5	0.875	1	54	28	1.31
14	2.243	13.72	23	71	19	14	1.273	1.936	113	59	2.425
15	2.26	9.324	18	60	14	11	1.185	1.971	88	46	2.103
16	2.396	20.345	21	73	14	18	1.16	1.868	99	52	2.483
17	2.396	11.627	21	73	14	18	1.16	1.868	99	52	2.33
18	2.369	11.204	14	48	8	10	0.853	0.722	58	28	1.068
19	3.1	21.175	26	102	22	29	2.187	2.231	122	70	5.452
20	3.1	20.675	26	102	22	29	2.187	2.231	122	70	5.858
21	2.45	24.92	38	158	22	18	1.029	1.506	171	88	4.873
22	2.453	23.917	41	103	18	17	0.769	0.918	166	85	4.379
23	2.512	28.931	23	102	18	27	1.124	1.855	110	58	2.383
24	2.512	31.085	23	102	18	27	1.124	1.855	110	58	2.573
25	2.32	11.61	17	64	12	11	1.185	1.522	80	42	2.073
26	2.396	17.859	16	70	13	17	0.845	1.322	73	36	1.258
27	2.437	20.001	20	80	16	18	1.16	1.936	95	50	2.717
28	2.437	20.893	20	80	16	18	1.16	1.936	95	50	2.695
29	3.631	30.981	25	146	24	42	1.888	1.959	118	68	5.733
30	2.26	11.025	18	60	14	11	1.185	1.971	88	46	2.311
31	3.376	29.883	24	96	18	37	1.859	1.936	109	63	4.126
32	2.437	20.222	20	80	16	18	1.16	1.936	95	50	2.941
33	2.437	20.893	20	80	16	18	1.16	1.936	95	50	2.695
34	3.033	15.472	22	72	16	28	1.922	1.971	98	57	4.649
35	2.437	20.001	20	80	16	18	1.16	1.936	95	50	2.717
36	2.243	16.093	23	71	19	14	1.273	1.936	113	59	2.641
37	2.26	10.108	18	60	14	11	1.185	1.971	88	46	2.311
38	2.156	10.238	16	44	10	10	0.853	1	74	35	1.073
39	2.156	10.914	16	44	10	10	0.853	1	74	35	1.073
40	2.437	18.381	20	80	16	18	1.16	1.936	95	50	2.483
41	2.044	19.068	23	86	16	18	1.126	1.959	114	55	1.464
42	2.106	10.81	15	64	13	11	1.185	1.571	80	39	1.073
43	2.26	10.108	18	60	14	11	1.185	1.971	88	46	2.311
44	2.26	9.965	18	60	14	11	1.185	1.971	88	46	2.311
45	2.481	20.27	19	87	18	18	1.16	1.981	97	51	2.382
46	2.394	19.231	20	84	18	18	1.16	1.936	99	52	2.382
47	2.362	20.311	22	66	14	18	1.16	1.936	99	52	2.382
48	2.362	18.58	22	66	14	18	1.16	1.936	99	52	2.382
49	2.26	9.616	18	60	14	11	1.185	1.971	88	46	2.311
50	2.437	15.124	20	80	16	18	1.16	1.936	95	50	2.3

*J*, Balaban-like index; *TIE*, E-state topological parameter; *UNIP*, unipolarity; *CENT*, centralization; *VAR*, variation; *BAC*, Balaban centric index; *LOP*, lopping centric index; *ICR*, radial centric; *CSI*, eccentric connectivity index; *ECC*, eccentricity; *PHI*, Kier flexibility index

### Docking studies on sterol carrier protein-2 (SCP-2)

The mechanism of action of the larvicidal and repellent activity exerted by EOs and their constituents is not fully described. Inhibition of the acetylcholinesterase (AChE) enzyme has been frequently proposed, a similar neurotoxic effect produced by organophosphorus and carbamate incesticides [69, 70]. Similar results have been reported when flies and cockroaches are exposed to eugenol and α-terpineol [71]. However, some authors agree that in most cases there is no relationship between inhibition of AChE and larvicidal effects of terpenes and derivatives [72, 73].

Priestley et al. proposes that EOs and their constituents act on GABA receptors, as indicated by their results when exposing *Drosophila melanogaster* to thymol [74]. In addition, Kumar *et al.* have reported that terpenes present in *Calotropis gigantea* have larvicidal activity due to the ability to block the sterol carrier protein (AeSCP-2) [75], which is partially responsible for intracellular cholesterol transport in insects [76]. The larvaes during the feeding step contain high concentrations of SCP-2 because they depend on exogenous sources of cholesterol for biosynthesis of steroid derivatives [77]. Therefore, compounds that can inhibit this protein have a high potential as vector control agents.

**Fig. 6:**
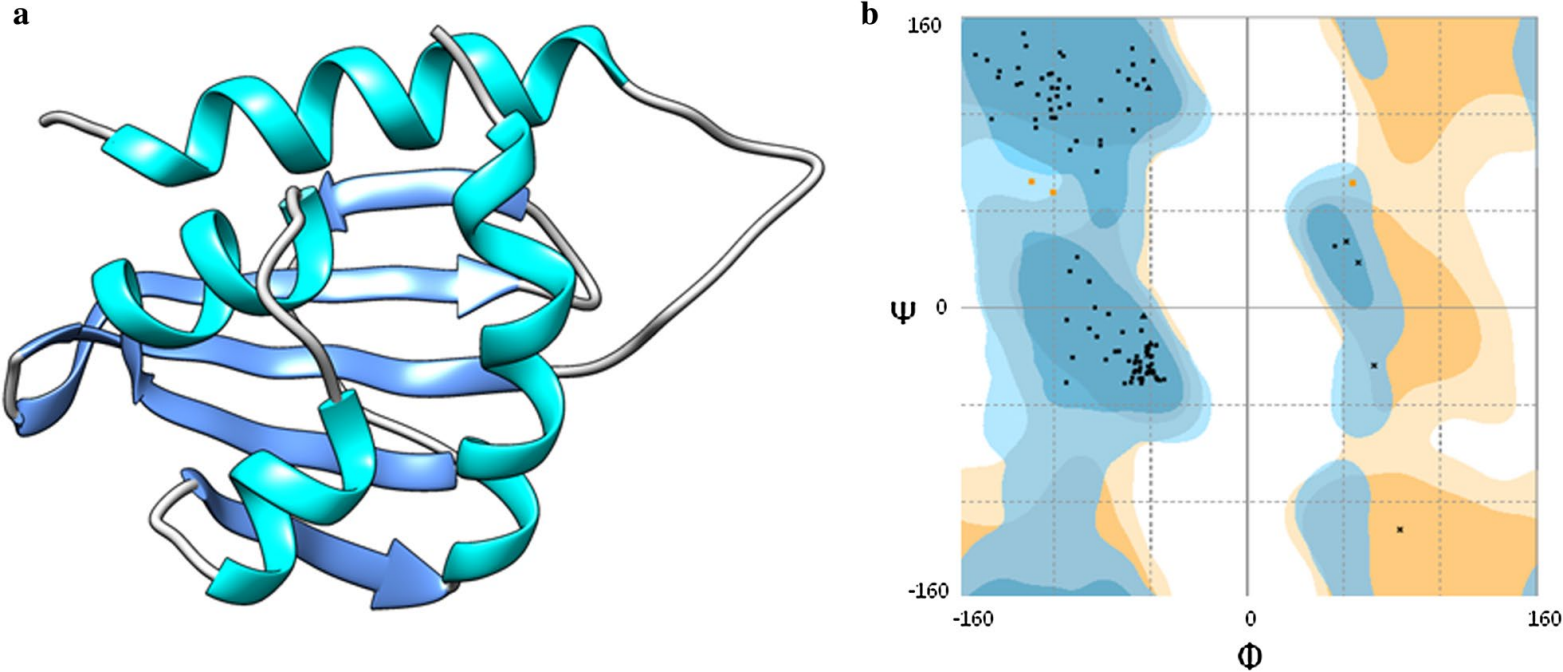
Results of the construction homology of the sterol carrier protein (SCP-2). **a** Model of sterol carrier protein (SCP-2). **b** Ramachandran plot corresponding for the model of SCP-2

With the purpose of estimating the interactions (theoretical affinity) of the evaluated compounds on sterol carrier protein (SCP-2) a docking study was carried out. The crystal structure of AeSCP-2 (*Aedes agypti* Sterol Carrier Protein ID-PBD: 1PZ4) was used for docking studies and to build its homologous enzyme from *Culex quinquefasciatus*. The SCP-2 sequence of *Culex quinquefasciatus* reported in the NCBI (GenBank: AA043438.1) presented a percentage of identity of 99.09% with AeSCP-2. Figure 6 shows the tridimensional (3D) model of SCP-2 and the corresponding Ramachandran plot used for evaluation. The analysis of the free energy values of the molecular interaction between the terpenes on SCP-2 enzyme showed that all the compounds bind strongly inside the active site with a similar binding mode; binding energies (ΔG) for each molecule are shown in Table 8.

**Table 8:** Docking results by SCP-2 from *Culex quinquefasciatus*

	Molecules	ΔG (kcal)	Interaction with amino acids
1	*p*-Anisaldehyde	− 5.72	N23, R24, Q25, V26, L102, F105
2	Canphor	− 5.86	L16, Q25,V26
3	3-Carene	− 6.17	I19, N23, R24, Q25, V26
4	Carvacrol	− 6.88	I19, R24, Q25, V26, L48, L102, F105
5	Carveol	− 5.22	R24, Q25, V26, L102, F105
6	Carvomenthol	− 5.69	I19, R24, Q25, V26, Q25
7	(+)-Carvone	− 6.62	R15, I19, D20, R24, N23, Q25, V26
8	Carvotanacetol	− 5.32	I19, R24, Q25, V26, Q25
9	*β*-Caryophyllene	− 7.87	R15, L16, I19, V26, L48, L102, F105
10	Citronellal	− 4.16	I19, D20, N23, R24, Q25, V26
11	*β*-Citronellol	− 5.29	I19, D20, N23, R24, Q25, V26, L48
12	*m*-Cresol	− 6.26	I19, R24*, Q25*, F105
13	*o*-Cresol	− 6.11	I19, R24, Q25, F105
14	Cuminaldehyde	− 5.72	N23, R24, Q25, V26, L48
15	*p*-Cymene	− 5.28	D20, N23, R24, Q25, V26
16	t-Dihydrocarvone	− 5.97	R15*, I19, D20, R24, N23, Q25, V26, F105
17	3,4-Dimethylcumene	− 5.22	D20, N23, R24, Q25, V26
18	Eucalyptol	− 5.03	R15, L16, L102
19	Geranial	− 5.96	I19, D20, N23, R24, Q25, V26
20	Geraniol	− 5.96	I19, D20, N23, R24, Q25, V26, L48, L102, F105
21	Germacrene-D	− 7.65	R15, L16, I19, V26, L48, L102, F105
22	α-Humulene	− 7.87	R15, L16, I19, V26, L48, L102
23	Hydrocarvone	− 5.72	I19, D20, R24, N23, Q25, V26
24	Hydrodihydrocarvone	− 5.81	R15, I19, D20, R24, N23, Q25, V26
25	3-Isopropylphenol	− 5.22	D20, N23, R24, Q25, V26
26	Isoborneol	− 5.21	R15, L16, L102
27	Isopulegol	− 6.26	I19, D20, R24, N23, Q25, V26, L48, L102
28	t-Isopulegone	− 6.44	R15*, I19, D20, R24, N23, Q25, V26, L48
29	Lavandullol	− 4.72	D20, N23, R24, Q25
30	Limonene	− 5.81	I19, N23, R24, Q25, V26, L48, L102
31	Linalool	− 5.76	I19, D20, N23, R24, Q25, V26
32	Menthol	− 5.69	I19, R24, Q25, V26, Q25
33	Menthone	− 5.51	R15, I19, R24, N23, Q25, V26
34	Myrcene	− 6.05	I19, N23, R24, Q25, L102, F105
35	Neoisopulegol	− 6.34	I19, N23, R24, Q25, V26
36	(− )-Perillaldehyde	− 5.95	R15, L16, I19, N23, R24, Q25, V26
37	Phellandrene	− 5.1	D20, N23, R24, Q25, V26
38	*α*-Pinene	− 5.85	R15, I19, N23, R24, Q25, V26, L48
39	*β*-Pinene	− 5.96	R15, I19, N23, R24, Q25, V26, L48
40	(+)-Pulegone	− 6.51	R15, I19, N23, R24, Q25, V26
41	Rotundifolone	− 6.33	I19, R24, Q25, V26, L48
42	Sabinene	− 5.5	I19, N23, R24,Q25, V26
43	α-Terpinene	− 6.76	I19, N23, R24, V26, L48
44	γ-Terpinene	− 6.85	I19, N23, R24, Q25, V26, L48
45	*4*-Terpineol	− 5.77	R15, I19, R24, V26, L102
46	*α*-Terpineol	− 5.46	I19, R24, V26, L102
47	*β*-Terpineol	− 5.13	I19, D20, R24, N23, Q25, V26
48	*γ*-Terpineol	− 5.14	I19, D20, R24, N23, Q25, V26
49	Terpinolene	− 6.01	I19, R24, V26, L48, L102, F105
50	Thymol	− 6.66	I19, D20, N23, R24, Q25, V26, L48

* Hydrogen bonds interaction

Results showed that monoterpenes and monoterpenoids with the highest larvicidal activity were also the compounds with better binding energy values, being carvacrol the most active followed by α-terpinene and terpinolene. Another important observation is that monoterpenes and monoterpenoides with the highest larvicidal activity are capable of interact with the Phe105 residue.

**Fig. 7:**
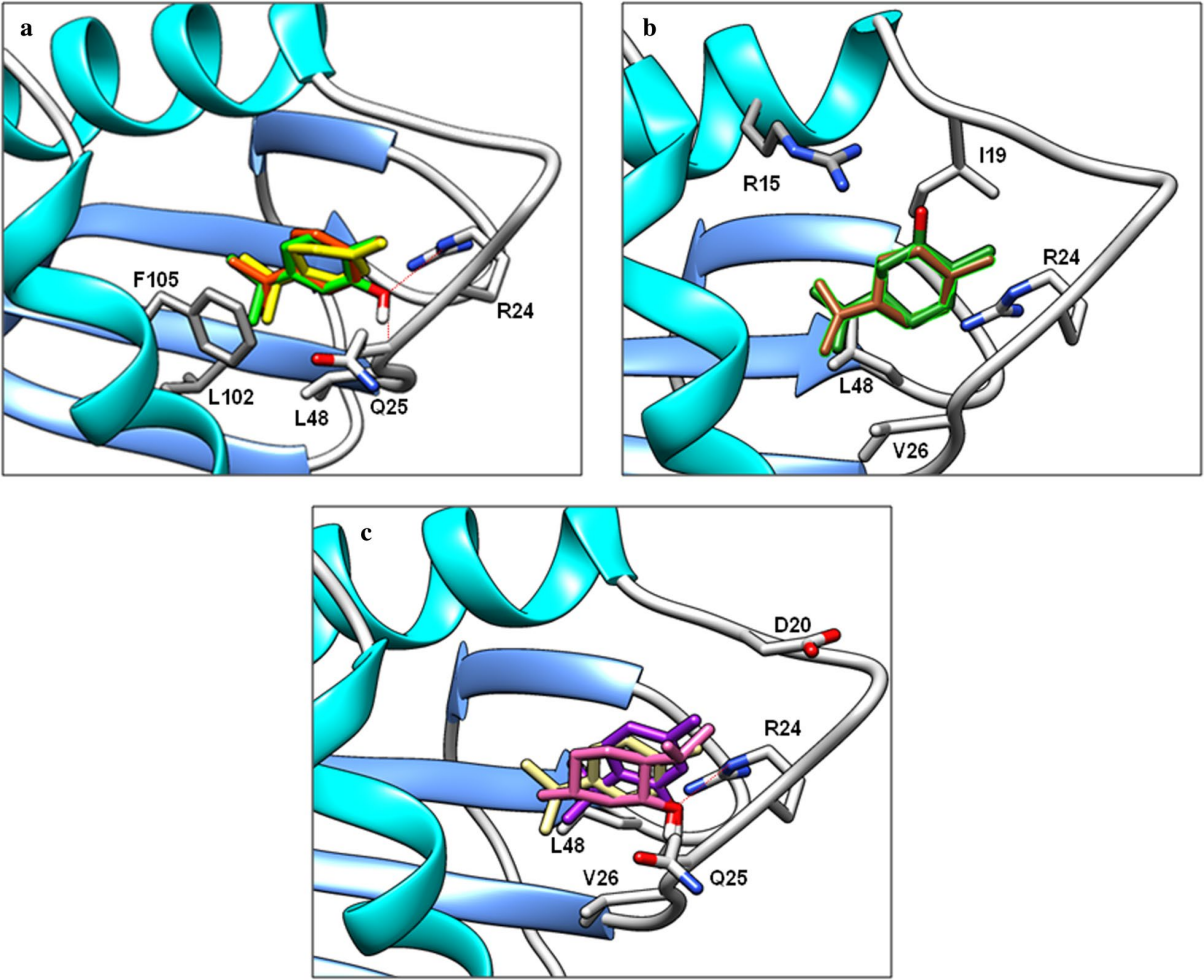
Interaction of cyclic terpenes and terpenoids with SCP-2. **a** Interaction of carvacrol (green), α-Terpinene (orange) and terpinolene (yellow). **b** Interaction of limonene (brown) and carvona (green). **c** Interaction of *p*-cimene (yellow), menthol (pink) and thymol (purple)

All cyclic terpenes and cyclic terpenoids interact with Arg24 and Val26 by hydrophobic interactions; only terpinene, terpinolene and carvacrol have interaction with the Phe105 residue. In these compounds, the greater number of π conjugated bonds, provides better interaction with SCP-2 (Fig. 7a). Carvone interacts to a lesser extent than limonene with the SCP-2 protein, since the keto group present in carvone makes the molecule more hydrophilic and therefore does not interact with and Leu48 residues Leu102, as does limonene (Fig. 7b). Results agree with QSAR descriptors related to their poor biological activity.

The relevance of the phenol group is observed when the binding energies of cymene, menthol, thymol and carvacrol are compared. Cymene binding energy is − 5.28 kcal, while menthol is − 5.69 kcal, this energy difference can be attributed to the hydroxyl group; on the other hand, thymol has a binding energy of − 6.66 kcal, which shows that the phenolic group is also important. This characteristics are also observed when comparing the bonding energy of carvomenthol and carvacrol. These results are consistent with the QSAR models also included in this work. The structural difference between the aromatic ring present in thymol and menthol without π bonds, generates a change in the arrangement of the later in the SCP-2 protein active site. It can be observed that the larger aliphatic chain in *para* position of cymene and thymol is in the direction of Phe105 residue, but does not interact with it, while the menthol is in the opposite position; however the hydroxyl group is kept in the same coordinates as for thymol (Fig. 7c). This is because the hydroxyl group of thymol and menthol are capable of forming hydrogen bonds with the amino group of Arg24 residue.

The position of the hydroxyl group in the phenolic group is also relevant. The hydroxyl group in the *meta* position of carvacrol leaves more exposed to larger aliphatic chain, which interacts with the Phe105 residue; the results is an increased biological activity as well as a more favorable binding energy as compared to thymol. The hydroxyl group of carvacrol can form hydrogen bonds with the amino group in Arg24 and with the amino group of the peptide bond between Gln25 and Val26 residues. The isopropyl group, on the other hand, also plays a fundamental role in the recognition of monoterpenes; for example, m-cresol and *o*-cresol, does not have the isopropyl residue and have no affinity on the SCP-2. This observation also agrees with the QSAR models, which propose that *nCt* are important in biological activity.

The results on acyclic terpenes denote the importance of π bonds despite not being aromatic moieties. Citronellol, the molecule with lower number of π bonds, is an acyclic terpene less able to interact with SCP-2 and is also the molecule with lower larvicidal activity. On the other hand, myrcene has the highest number of π bonds, presented the highest larvicidal activity and is also the best to interact with SCP-2. Geraniol and myrcene are the acyclic terpenes with the higher larvicidal activity and both interact with the Phe105 residue (Additional file 1: Figure S2). All acyclic terpenes, except those with ketone groups, are capable of interact with residues Ile19, Asn23, Arg24 and Gln25. Geraniol has the ability to form a hydrogen bond with the amino group of the backbone between the Ile19 and Asn20.

Anisaldehyde presented a binding energy of − 5.72 kcal/mol and was able to interact with the Phe105 residue and form a hydrogen bond with the amino group of Arg24 (Additional file 1: Figure S3a). The cuminaldehyde does not interact with the Phe105 residue and was not able to form hydrogen bonds. Sesquiterpenes presented the highest affinity on the SCP-2 active site, presented interactions with the Phe105 residue and with the hydrophobic pocket (Additional file 1: Figure S3b).

### Conclusions

The larvicidal activity of terpenes and terpenoids was analyzed by LC_50_ determination for different stairs of *Culex quinquefasciatus* Say. The description of the molecular properties and the structural characteristics responsible for larvicidal activity of the tested compounds, were used for the development of mathematical models of structure–property–activity relationship. The docking studies were able to show that molecular and structural descriptors provide evidence of SCP-2 as a possible biological target, an important protein in cholesterol and fatty acid catabolism, which cleaves the 3-oxoacyl-CoAs of methyl-branched fatty acid and bile acid intermediates. However experimental studies should be conducted to elucidate this effect.

## Additional file

**Additional file 1.** Additional tables and figures.
