# Supplementary material for: In vitro and in silico studies of terpenes, terpenoids and related compounds with larvicidal and pupaecidal activity against Culex quinquefasciatus Say (Diptera: Culicidae)
Source: Chem Cent J. 2018 May 10;12:53. doi: 10.1186/s13065-018-0425-2 (PMC5945571; doi:10.1186/s13065-018-0425-2)

**Additional file 1: Table S1.** Experimental and predicted LogLC_50_ values for QSAR models

|  | | ***Model 1*** | | ***Model 3*** | | ***Model 5*** | |
| --- | --- | --- | --- | --- | --- | --- | --- |
| **Compound** | **Log Exp.** | **Predicted** | **Residual** | **Predicted** | **Residual** | **Predicted** | **Residual** |
| 1 | 1.27 | 1.44 | 0.17 | 1.45 | 0.18 | 1.23 | -0.04 |
| 2 | 1.41 | 2.12 | 0.71 | 1.73 | 0.32 | *Excluded* | |
| 3 | 1.41 | 1.39 | -0.02 | 1.45 | 0.04 | *Excluded* | |
| 4 | 0.89 | 1.08 | 0.19 | 1.38 | 0.49 | 1.06 | 0.17 |
| 5 | 2.02 | 2.05 | 0.03 | 1.85 | -0.17 | 2.1 | 0.08 |
| 6 | 2.34 | 2.74 | 0.4 | 2.62 | 0.28 | 2.64 | 0.3 |
| 7 | 2.18 | 2.14 | -0.04 | 1.85 | -0.33 | 2.36 | 0.18 |
| 8 | 2.3 | 2.38 | 0.08 | 2.08 | -0.22 | 2.37 | 0.07 |
| 9 | 1.67 | 1.29 | -0.38 | *Excluded* | | *Excluded* | |
| 10 | 2.1 | 2.07 | -0.03 | 2.08 | -0.02 | *Excluded* | |
| 11 | 1.98 | 1.87 | -0.11 | 2.08 | 0.1 | *Excluded* | |
| 12 | 1.78 | 1.37 | -0.41 | 1.61 | -0.17 | 1.76 | -0.02 |
| 13 | 1.74 | 1.42 | -0.32 | 1.61 | -0.13 | 1.79 | 0.05 |
| 14 | 1.38 | 1.43 | 0.05 | 1.2 | -0.18 | 1 | -0.38 |
| 15 | 1.38 | 1.42 | 0.04 | 1.36 | -0.02 | 1.59 | 0.21 |
| 16 | 2.56 | 2.4 | -0.16 | 2.39 | -0.17 | 2.63 | 0.07 |
| 17 | 1.68 | 1.02 | -0.66 | 1.61 | -0.07 | 1.43 | -0.25 |
| 18 | 1.65 | 2.1 | 0.45 | 1.48 | -0.17 | *Excluded* | |
| 19 | 1.73 | 1.78 | 0.05 | 1.53 | -0.2 | *Excluded* | |
| 20 | 1.31 | 1.63 | 0.32 | 1.53 | 0.22 | *Excluded* | |
| 21 | 1.66 | 2.11 | 0.45 | Excluded | | *Excluded* | |
| 22 | 2.01 | 1.68 | -0.33 | Excluded | | *Excluded* | |
| 23 | 3.17 | 2.47 | -0.7 | 2.88 | -0.29 | 2.79 | -0.38 |
| 24 | 3.21 | 2.63 | -0.58 | 2.88 | -0.33 | 3.23 | 0.02 |
| 25 | 1.36 | 1.43 | 0.07 | 1.61 | 0.25 | 1 | -0.36 |
| 26 | 1.99 | 2.01 | 0.02 | 1.73 | -0.26 | *Excluded* | |
| 27 | 2.47 | 2.32 | -0.15 | 2.39 | -0.08 | 2.31 | -0.16 |
| 28 | 2.73 | 2.76 | 0.03 | 2.62 | -0.11 | 2.9 | 0.17 |
| 29 | 1.75 | 1.53 | -0.22 | 1.85 | 0.1 | *Excluded* | |
| 30 | 1.44 | 1.85 | 0.41 | 1.59 | 0.15 | 1.51 | 0.07 |
| 31 | 1.49 | 1.89 | 0.4 | 1.76 | 0.27 | *Excluded* | |
| 32 | 2.61 | 2.65 | 0.04 | 2.62 | 0.01 | 2.67 | 0.06 |
| 33 | 2.71 | 2.76 | 0.05 | 2.62 | -0.09 | 2.9 | 0.19 |
| 34 | 1.28 | 1.64 | 0.36 | 1.28 | 0 | *Excluded* | |
| 35 | 2.74 | 2.2 | -0.54 | 2.39 | -0.35 | 2.35 | -0.39 |
| 36 | 2.06 | 2.03 | -0.03 | 1.85 | -0.21 | 0.01 | -2.05 |
| 37 | 2.74 | 1.77 | -0.97 | 2.42 | -0.32 | 1.48 | -1.26 |
| 38 | 1.41 | 1.39 | -0.02 | 1.38 | -0.03 | *Excluded* | |
| 39 | 1.39 | 1.59 | 0.2 | 1.69 | 0.3 | *Excluded* | |
| 40 | 2.27 | 2.32 | 0.05 | 2.16 | -0.11 | 2.33 | 0.06 |
| 41 | 1.8 | 2.46 | 0.66 | 2.42 | 0.62 | 1.8 | 0 |
| 42 | 1.77 | 1.76 | -0.01 | 1.69 | -0.08 | *Excluded* | |
| 43 | 1.13 | 1.89 | 0.76 | 1.28 | 0.15 | 1.48 | 0.35 |
| 44 | 1.75 | 1.91 | 0.16 | 1.28 | -0.47 | 1.33 | -0.42 |
| 45 | 1.99 | 2.21 | 0.22 | 2.08 | 0.09 | 2.1 | 0.11 |
| 46 | 1.99 | 2.17 | 0.18 | 2.08 | 0.09 | 2.14 | 0.15 |
| 47 | 2.03 | 2.17 | 0.14 | 2.39 | 0.36 | 2.12 | 0.09 |
| 48 | 2.02 | 2.01 | -0.01 | 2.16 | 0.14 | 1.81 | -0.21 |
| 49 | 1.27 | 1.73 | 0.46 | 1.36 | 0.09 | 1.19 | -0.08 |
| 50 | 1.09 | 1 | -0.09 | 1.39 | 0.3 | 0.95 | -0.14 |

**Additional file 1: Table S2.** Constitutional descriptors calculated for fifty compounds evaluated.

| **Mol.** | ***MW*** | ***Sv*** | ***Se*** | ***Sp*** | ***Mv*** | ***Me*** | ***Mp*** | ***nAT*** | ***nSK*** | ***nBT*** | ***nBO*** | ***nBM*** | ***SCBO*** |
| --- | --- | --- | --- | --- | --- | --- | --- | --- | --- | --- | --- | --- | --- |
| 1 | 136.16 | 11.41 | 18.19 | 11.95 | 0.63 | 1.01 | 0.66 | 18 | 10 | 18 | 10 | 7 | 14 |
| 2 | 152.26 | 15.29 | 26.4 | 16.55 | 0.57 | 0.98 | 0.61 | 27 | 11 | 28 | 12 | 1 | 13 |
| 3 | 136.26 | 14.78 | 25.07 | 16.09 | 0.57 | 0.96 | 0.62 | 26 | 10 | 27 | 11 | 1 | 12 |
| 4 | 150.24 | 14.7 | 24.51 | 15.78 | 0.59 | 0.98 | 0.63 | 25 | 11 | 25 | 11 | 6 | 14 |
| 5 | 152.26 | 15.29 | 26.4 | 16.55 | 0.57 | 0.98 | 0.61 | 27 | 11 | 27 | 11 | 2 | 13 |
| 6 | 156.3 | 16.49 | 30.16 | 18.07 | 0.53 | 0.97 | 0.58 | 31 | 11 | 31 | 11 | 0 | 11 |
| 7 | 150.24 | 14.7 | 24.51 | 15.78 | 0.59 | 0.98 | 0.63 | 25 | 11 | 25 | 11 | 3 | 14 |
| 8 | 154.28 | 15.89 | 28.28 | 17.31 | 0.55 | 0.98 | 0.6 | 29 | 11 | 29 | 11 | 1 | 12 |
| 9 | 190.36 | 20.58 | 34.72 | 22.38 | 0.57 | 0.96 | 0.62 | 36 | 14 | 37 | 15 | 2 | 17 |
| 10 | 154.28 | 15.89 | 28.28 | 17.31 | 0.55 | 0.98 | 0.6 | 29 | 11 | 28 | 10 | 2 | 12 |
| 11 | 156.3 | 16.49 | 30.16 | 18.07 | 0.53 | 0.97 | 0.58 | 31 | 11 | 30 | 10 | 1 | 11 |
| 12 | 108.15 | 9.9 | 15.86 | 10.5 | 0.62 | 0.99 | 0.66 | 16 | 8 | 16 | 8 | 6 | 11 |
| 13 | 108.15 | 9.9 | 15.86 | 10.5 | 0.62 | 0.99 | 0.66 | 16 | 8 | 16 | 8 | 6 | 11 |
| 14 | 148.22 | 14.1 | 22.63 | 15.02 | 0.61 | 0.98 | 0.65 | 23 | 11 | 23 | 11 | 7 | 15 |
| 15 | 134.24 | 14.18 | 23.19 | 15.33 | 0.59 | 0.97 | 0.64 | 24 | 10 | 24 | 10 | 6 | 13 |
| 16 | 152.26 | 15.29 | 26.4 | 16.55 | 0.57 | 0.98 | 0.61 | 27 | 11 | 27 | 11 | 2 | 13 |
| 17 | 148.27 | 15.78 | 26.07 | 17.09 | 0.58 | 0.97 | 0.63 | 27 | 11 | 27 | 11 | 6 | 14 |
| 18 | 138.28 | 15.38 | 26.95 | 16.85 | 0.55 | 0.96 | 0.6 | 28 | 10 | 29 | 11 | 0 | 11 |
| 19 | 152.26 | 15.29 | 26.4 | 16.55 | 0.57 | 0.98 | 0.61 | 27 | 11 | 26 | 10 | 3 | 13 |
| 20 | 154.28 | 15.89 | 28.28 | 17.31 | 0.55 | 0.98 | 0.6 | 29 | 11 | 28 | 10 | 2 | 12 |
| 21 | 204.39 | 22.17 | 37.6 | 24.14 | 0.57 | 0.96 | 0.62 | 39 | 15 | 39 | 15 | 3 | 18 |
| 22 | 204.39 | 22.17 | 37.6 | 24.14 | 0.57 | 0.96 | 0.62 | 39 | 15 | 39 | 15 | 3 | 18 |
| 23 | 168.26 | 15.81 | 27.72 | 17 | 0.56 | 0.99 | 0.61 | 28 | 12 | 28 | 12 | 2 | 14 |
| 24 | 170.28 | 16.4 | 29.61 | 17.76 | 0.55 | 0.99 | 0.59 | 30 | 12 | 30 | 12 | 1 | 13 |
| 25 | 136.21 | 13.1 | 21.63 | 14.02 | 0.6 | 0.98 | 0.64 | 22 | 10 | 22 | 10 | 6 | 13 |
| 26 | 154.28 | 15.89 | 28.28 | 17.31 | 0.55 | 0.98 | 0.6 | 29 | 11 | 30 | 12 | 0 | 12 |
| 27 | 154.28 | 15.89 | 28.28 | 17.31 | 0.55 | 0.98 | 0.6 | 29 | 11 | 29 | 11 | 1 | 12 |
| 28 | 154.28 | 15.89 | 28.28 | 17.31 | 0.55 | 0.98 | 0.6 | 29 | 11 | 29 | 11 | 1 | 12 |
| 29 | 168.31 | 17.49 | 31.16 | 19.07 | 0.55 | 0.97 | 0.6 | 32 | 12 | 31 | 11 | 2 | 13 |
| 30 | 136.26 | 14.78 | 25.07 | 16.09 | 0.57 | 0.96 | 0.62 | 26 | 10 | 26 | 10 | 2 | 12 |
| 31 | 154.28 | 15.89 | 28.28 | 17.31 | 0.55 | 0.98 | 0.6 | 29 | 11 | 28 | 10 | 2 | 12 |
| 32 | 156.3 | 16.49 | 30.16 | 18.07 | 0.53 | 0.97 | 0.58 | 31 | 11 | 31 | 11 | 0 | 11 |
| 33 | 154.28 | 15.89 | 28.28 | 17.31 | 0.55 | 0.98 | 0.6 | 29 | 11 | 29 | 11 | 1 | 12 |
| 34 | 136.26 | 14.78 | 25.07 | 16.09 | 0.57 | 0.96 | 0.62 | 26 | 10 | 25 | 9 | 3 | 12 |
| 35 | 154.28 | 15.89 | 28.28 | 17.31 | 0.55 | 0.98 | 0.6 | 29 | 11 | 29 | 11 | 1 | 12 |
| 36 | 150.24 | 14.7 | 24.51 | 15.78 | 0.59 | 0.98 | 0.63 | 25 | 11 | 25 | 11 | 3 | 14 |
| 37 | 136.26 | 14.78 | 25.07 | 16.09 | 0.57 | 0.96 | 0.62 | 26 | 10 | 26 | 10 | 2 | 12 |
| 38 | 136.26 | 14.78 | 25.07 | 16.09 | 0.57 | 0.96 | 0.62 | 26 | 10 | 27 | 11 | 1 | 12 |
| 39 | 136.26 | 14.78 | 25.07 | 16.09 | 0.57 | 0.96 | 0.62 | 26 | 10 | 27 | 11 | 1 | 12 |
| 40 | 152.26 | 15.29 | 26.4 | 16.55 | 0.57 | 0.98 | 0.61 | 27 | 11 | 27 | 11 | 2 | 13 |
| 41 | 166.24 | 15.21 | 25.84 | 16.24 | 0.58 | 0.99 | 0.62 | 26 | 12 | 27 | 13 | 2 | 15 |
| 42 | 136.26 | 14.78 | 25.07 | 16.09 | 0.57 | 0.96 | 0.62 | 26 | 10 | 27 | 11 | 1 | 12 |
| 43 | 136.26 | 14.78 | 25.07 | 16.09 | 0.57 | 0.96 | 0.62 | 26 | 10 | 26 | 10 | 2 | 12 |
| 44 | 136.26 | 14.78 | 25.07 | 16.09 | 0.57 | 0.96 | 0.62 | 26 | 10 | 26 | 10 | 2 | 12 |
| 45 | 154.28 | 15.89 | 28.28 | 17.31 | 0.55 | 0.98 | 0.6 | 29 | 11 | 29 | 11 | 1 | 12 |
| 46 | 154.28 | 15.89 | 28.28 | 17.31 | 0.55 | 0.98 | 0.6 | 29 | 11 | 29 | 11 | 1 | 12 |
| 47 | 154.28 | 15.89 | 28.28 | 17.31 | 0.55 | 0.98 | 0.6 | 29 | 11 | 29 | 11 | 1 | 12 |
| 48 | 154.28 | 15.89 | 28.28 | 17.31 | 0.55 | 0.98 | 0.6 | 29 | 11 | 29 | 11 | 1 | 12 |
| 49 | 136.26 | 14.78 | 25.07 | 16.09 | 0.57 | 0.96 | 0.62 | 26 | 10 | 26 | 10 | 2 | 12 |
| 50 | 150.24 | 14.7 | 24.51 | 15.78 | 0.59 | 0.98 | 0.63 | 25 | 11 | 25 | 11 | 6 | 14 |

***MW*** = molecular weight; ***Sv*** = sum of atomic van der Waals volumes (scaled on Carbon atom); ***Se*** = sum of atomic Sanderson electronegativities (scaled on Carbon atom); ***Sp*** = sum of atomic polarizabilities (scaled on Carbon atom); ***Mv*** = mean atomic van der Waals volume (scaled on Carbon atom); ***Me*** = mean atomic Sanderson electronegativity (scaled on Carbon atom); ***Mp*** = mean atomic polarizability (scaled on Carbon atom); ***nAT*** = number of atoms; ***nSK*** = number of non-H atoms; ***nBT*** = number of bonds; ***nBO*** = number of non-H bonds; ***nBM*** = number of multiple bonds; ***SCBO*** = sum of conventional bond orders (H-depleted).

**Additional file 1: Table S3.** Experimental and predicted LogLC_50_ values for QPAR models

|  | | ***Model 1*** | | ***Model 3*** | | ***Model 5*** | |
| --- | --- | --- | --- | --- | --- | --- | --- |
| **Compound** | **Log Exp.** | **Predicted** | **Residual** | **Predicted** | **Residual** | **Predicted** | **Residual** |
| 1 | 1.27 | 1.41 | 0.14 | 1.31 | 0.04 | 1.44 | 0.17 |
| 2 | 1.41 | 2 | 0.59 | 1.9 | 0.49 | 1.44 | 0.03 |
| 3 | 1.41 | 1.54 | 0.13 | 1.5 | 0.09 | 1.44 | 0.03 |
| 4 | 0.89 | 1.27 | 0.38 | 1.32 | 0.43 | 1.39 | 0.5 |
| 5 | 2.02 | 1.9 | -0.12 | 2.12 | 0.1 | 2.18 | 0.16 |
| 6 | 2.34 | 2.62 | 0.28 | 2.45 | 0.11 | 2.75 | 0.41 |
| 7 | 2.18 | 1.84 | -0.34 | 1.68 | -0.5 | 2.31 | 0.13 |
| 8 | 2.3 | 2.19 | -0.11 | 1.6 | -0.7 | 2.4 | 0.1 |
| 9 | 1.67 | 1.3 | -0.37 | *Excluded* | | *Excluded* | |
| 10 | 2.1 | 2.08 | -0.02 | 2.61 | 0.51 | *Excluded* | |
| 11 | 1.98 | 2.16 | 0.18 | 1.73 | -0.25 | *Excluded* | |
| 12 | 1.78 | 1.4 | -0.38 | 1.46 | -0.32 | 1.76 | -0.02 |
| 13 | 1.74 | 1.45 | -0.29 | 1.63 | -0.11 | 1.8 | 0.06 |
| 14 | 1.38 | 1.35 | -0.03 | 1.34 | -0.04 | 1.64 | 0.26 |
| 15 | 1.38 | 1.18 | -0.2 | 1.45 | 0.07 | 0.81 | -0.57 |
| 16 | 2.56 | 2.26 | -0.3 | 2.53 | -0.03 | 2.58 | 0.02 |
| 17 | 1.68 | 0.82 | -0.86 | 1.74 | 0.06 | 1.29 | -0.39 |
| 18 | 1.65 | 1.44 | -0.21 | 1.67 | 0.02 | 1.7 | 0.05 |
| 19 | 1.73 | 1.45 | -0.28 | 1.61 | -0.12 | *Excluded* | |
| 20 | 1.31 | 1.6 | 0.29 | 1.17 | -0.14 | *Excluded* | |
| 21 | 1.66 | 2.26 | 0.6 | Excluded | | *Excluded* | |
| 22 | 2.01 | 1.83 | -0.18 | Excluded | | *Excluded* | |
| 23 | 3.17 | 2.38 | -0.79 | 3.43 | 0.26 | 3.29 | 0.12 |
| 24 | 3.21 | 2.6 | -0.61 | 2.77 | -0.44 | 3.57 | 0.36 |
| 25 | 1.36 | 1.52 | 0.16 | 1.81 | 0.45 | 0.37 | -0.99 |
| 26 | 1.99 | 2.01 | 0.02 | 2.19 | 0.2 | *Excluded* | |
| 27 | 2.47 | 2.33 | -0.14 | 2.45 | -0.02 | 2.24 | -0.23 |
| 28 | 2.73 | 2.55 | -0.18 | 2.49 | -0.24 | 2.66 | -0.07 |
| 29 | 1.75 | 1.05 | -0.7 | 2.54 | 0.79 | *Excluded* | |
| 30 | 1.44 | 1.7 | 0.26 | 1.72 | 0.28 | 1.14 | -0.3 |
| 31 | 1.49 | 1.69 | 0.2 | 1.82 | 0.33 | *Excluded* | |
| 32 | 2.61 | 2.6 | -0.01 | 3.41 | 0.8 | 2.49 | -0.12 |
| 33 | 2.71 | 2.56 | -0.15 | 2.25 | -0.46 | 2.67 | -0.04 |
| 34 | 1.28 | 0.76 | -0.52 | 0.81 | -0.47 | *Excluded* | |
| 35 | 2.74 | 2.33 | -0.41 | 2.35 | -0.39 | 2.27 | -0.47 |
| 36 | 2.06 | 1.99 | -0.07 | 2.01 | -0.05 | 1.95 | -0.11 |
| 37 | 2.74 | 1.56 | -1.18 | 1.77 | -0.97 | 1.17 | -1.57 |
| 38 | 1.41 | 1.56 | 0.15 | 1.78 | 0.37 | 1.22 | -0.19 |
| 39 | 1.39 | 1.63 | 0.24 | 2.34 | 0.95 | 1.22 | -0.17 |
| 40 | 2.27 | 2.11 | -0.16 | 2.26 | -0.01 | 2.26 | -0.01 |
| 41 | 1.8 | 2.03 | 0.23 | 2.19 | 0.39 | 1.74 | -0.06 |
| 42 | 1.77 | 1.57 | -0.2 | 1.43 | -0.34 | 1.95 | 0.18 |
| 43 | 1.13 | 1.73 | 0.6 | 1.28 | 0.15 | 1.22 | 0.09 |
| 44 | 1.75 | 1.63 | -0.12 | 2.03 | 0.28 | 1.17 | -0.58 |
| 45 | 1.99 | 2.36 | 0.37 | 2.48 | 0.49 | 1.8 | -0.19 |
| 46 | 1.99 | 2.22 | 0.23 | 2.2 | 0.21 | 1.96 | -0.03 |
| 47 | 2.03 | 2.3 | 0.27 | 2.07 | 0.04 | 1.92 | -0.11 |
| 48 | 2.02 | 2.17 | 0.15 | 2.08 | 0.06 | 1.84 | -0.18 |
| 49 | 1.27 | 1.61 | 0.34 | 1.63 | 0.36 | 1.06 | -0.21 |
| 50 | 1.09 | 1.28 | 0.19 | 1.35 | 0.26 | 1.34 | 0.25 |

**Additional file 1: Figure S1.** The contour plots of LUMO orbitals of the most active molecules.


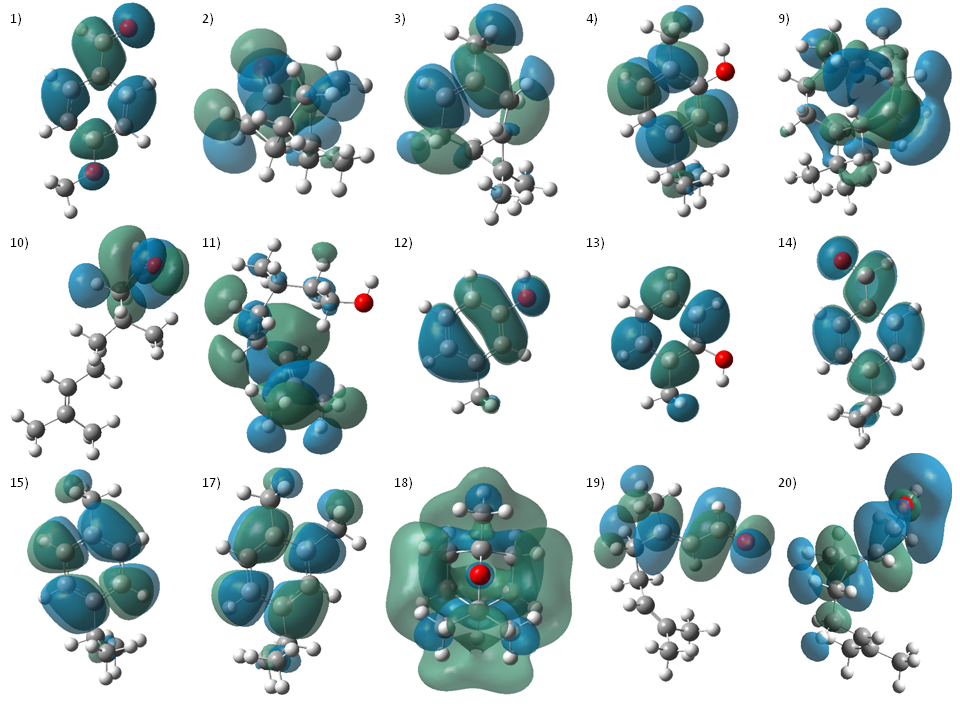

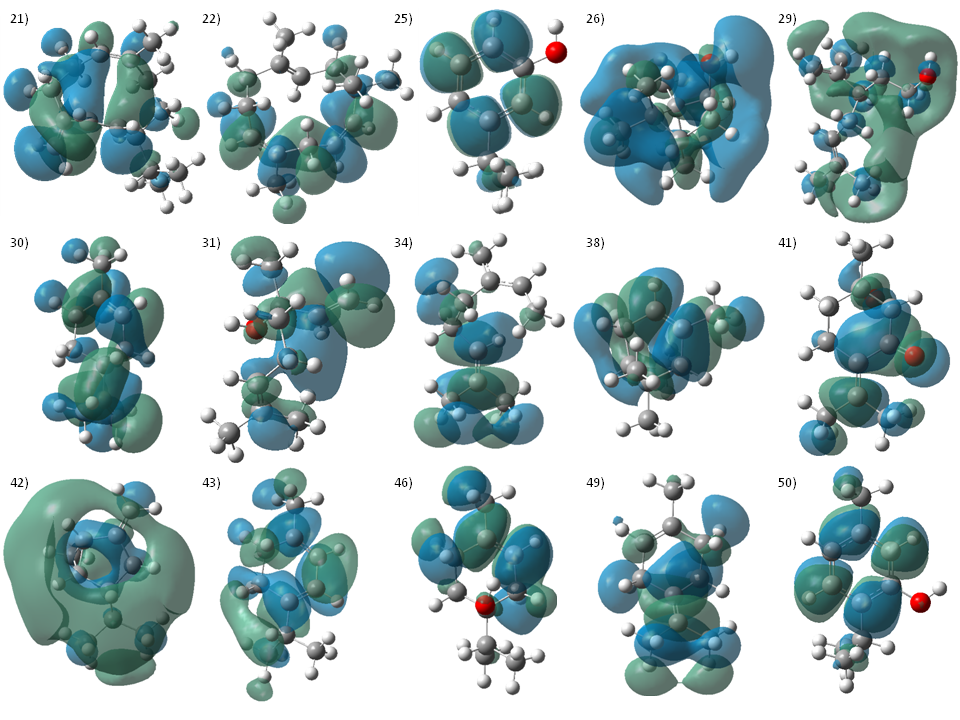


1) p-Anisaldehyde, 2) Canphor, 3) 3-Carene, 4) Carvacrol, 9) β-Caryophyllene, 10) Citronellal, 11) β-Citronellol, 12) m-cresol, 13) o-cresol, 14) Cuminaldehyde, 15) p-Cimene, 17) 3,4-Dimethylcumene, 18)Eucalyptol, 19) Geranial, 20) Geraniol, 21) Germacrene-D, 22) α-Humulene, 25) 3-Isopropylphenol, 26) Isoborneol, 29) Lavandullol, 30) Limonene, 31) Linalool, 34) Myrcene, 38) α-Pinene, 41) Rotundifolone, 42) Sabinene 43) α-Terpinene, 46) α-Terpineol, 48) γ-Terpineol, 49) Terpinolene, 50) Thymol

**Additional file 1: Figure S2.** Interaction of acyclic terpenes and terpenoids with SCP-2. a) Interaction of geraniol (pink) and myrcene (purple). b) Interaction of β-citronellol (green) and linalol (yellow).

**
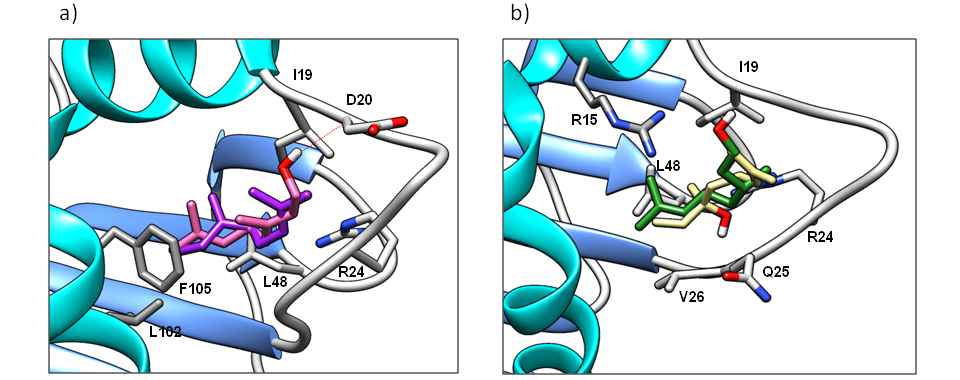
**

**Additional file 1: Figure S3.** Interaction of aldehydes and β-cariophylene with SCP-2. a) Interaction of anisaldehyde (green) and cuminaldehyde (yellow). b) Interaction of β-cariophylene (orange).


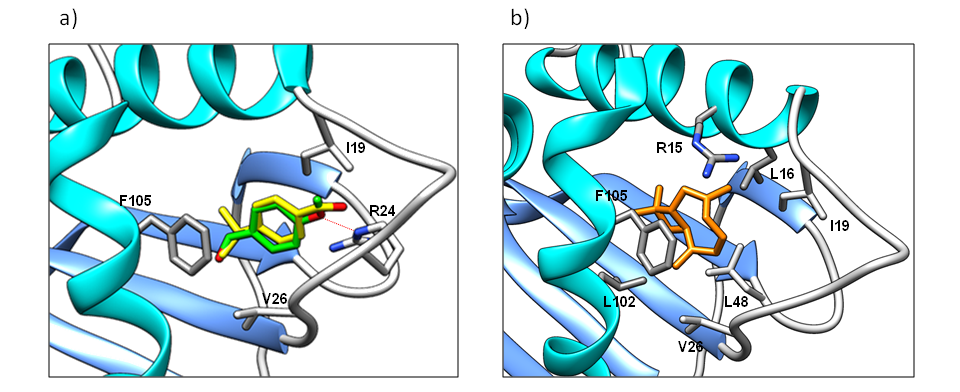

Supplement: Supplementary file 1 — Additional file 1. Additional tables and figures. [file 13065_2018_425_MOESM1_ESM.docx]
